# Genome-wide association mapping of bread wheat genotypes using yield and grain morphology-related traits under different environments

**DOI:** 10.3389/fgene.2022.1008024

**Published:** 2023-01-17

**Authors:** Hafiz Ghulam Muhu-Din Ahmed, Yawen Zeng, Muhammad Ahsan Khan, Muhammad Abdul Rehman Rashid, Muhammad Ameen, Ahmed Akrem, Amjad Saeed

**Affiliations:** ^1^ Department of Plant Breeding and Genetics, Faculty of Agriculture and Environment, The Islamia University of Bahawalpur, Bahawalpur, Pakistan; ^2^ Biotechnology and Germplasm Resources Institute, Yunnan Academy of Agricultural Sciences, Kunming, China; ^3^ Department of Plant Breeding and Genetics, University of Agriculture, Faisalabad, Pakistan; ^4^ Department of Agricultural Sciences, Government College University Faisalabad, Faisalabad, Pakistan; ^5^ Department of Bioinformatics and Biotechnology, Government College University Faisalabad, Faisalabad, Pakistan; ^6^ Department of Soil Science, Faculty of Agriculture and Environment, The Islamia University of Bahawalpur, Bahawalpur, Pakistan; ^7^ Institute of Botany, Bahauddin Zakariya University, Multan, Pakistan; ^8^ Institute of Forest Sciences Faculty of Agriculture and Environment, The Islamia University of Bahawalpur, Bahawalpur, Pakistan

**Keywords:** drought, heat, breeding, genome, chromosome, genotypes, wheat, yield

## Abstract

Depleting water resources and increasing global temperature due to climate change are major challenges to agriculture and food security worldwide. Deciphering the underlying mechanisms of traits contributing to grain development and yield is essential for the development of climate-resilient cultivars. Therefore, this study assessed 105 bread wheat genotypes grown under control, drought, and heat-stress conditions for two crop seasons and performed a genome-wide association study (GWAS) using a 90k SNP array. The genotypes showed significant trait differences under all environmental conditions. Highly significant variation was observed, with moderate (50.09%) to high (76.19%) heritability in the studied germplasms. The studied traits were all also significantly positively correlated. A total of 541 significant associations (*p ≤* 10^−3^) between marker and trait (MTAs) were observed after crossing the FDR <0.05 threshold for all traits. Among these, 195, 179, and 167 significant MTAs were detected under control, drought, and heat-stress conditions, respectively. Under the control and drought conditions, pleiotropic loci *BS00010616_51* and *BS00010868_51* were observed on chromosomes 7B and 1B situated at 186.24 cM and 35.47 cM, respectively. Pleiotropic loci *BS00010868_51*, *Kukri_c11154_1723*, and *Ex_c10068_1509* were identified on chromosomes 1B, 5B, and 2A, respectively, under control and heat stress conditions. A stable and consistent locus (*Excalibur_c20796_395*) on chromosome 7A, located at 372.34 cM, was also linked to grain morphology and yield-related attributes in control, drought, and heat-stress conditions. The results of the current study confirmed several previously reported MTAs for the traits under consideration and identified new MTAs under harsh climatic conditions. These SNPs will aid in the discovery of novel genes in wheat. SNPs showing significant associations may be used in marker-assisted selection and allow the development of drought- and heat-tolerant genotypes with high yields to address global food security concerns.

## Introduction

Wheat is a vital staple food in many countries worldwide. Overcoming the need and supply gap of food requires improving wheat yield against environmental stresses including drought and heat. The shape, texture, and size of wheat grains determine their economic worth. However, the genetic inheritance patterns of the morphological trait of wheat grain require further investigation. Wheat milling and baking quality are influenced by these traits, which include seed size, shape, length, and width, as well as grain sphericity (Ahamed et al., 2017). Among abiotic stresses, drought is the most significant global threat to sustainable wheat production. Drought decreases grain weight and yield per plant by inhibiting photosynthate transfer to the grains (Pinto et al., 2010). It stops metabolic processes, photosynthate synthesis, and translocation activities (Ahmed et al., 2019). Drought and heat stress have become more common, especially in wheat-growing areas worldwide, owing to changes in temperature and rainfall patterns. These stresses have a deleterious impact on wheat plant development and production. Water scarcity in developing countries has lowered wheat yield to 50–90% of their irrigated potential (Zampieri et al., 2017).

Wheat crops are not as water-intensive as rice and maize. However, water stress during specific vegetative and reproductive phases like tillering, jointing, booting, flowering, and grain-filling severely hamper crop yield. Although grain yield per plant (GYP) improvements have been made in irrigated-farmed regions, rainfed and water-stressed areas have seen far less success. Drought stress exacerbates the situation, with substantial production gaps between maximum productive regions and dryland farming (Qaseem et al., 2018).

Wheat is a cold-season crop, with an ideal daytime temperature for optimal growth of around 22 °C, followed by 16 °C at night. Temperature increases, especially around the flowering stage, drastically reduce grain number and size. Each degree Celsius beyond the optimal temperature results in a yield reduction of 3–4% (Ni et al., 2018). However, the worldwide average temperature increase is 0.18°C per decade (Hansen et al., 2012). Climate change is generating a rise in global average temperatures and the frequency of extreme weather events (Moriondo et al., 2011). From 1880 to 2012, the average temperature of the Earth’s surface increased by 0.85°C, a trend that is expected to continue (Guo et al., 2020). Therefore, investigation of the desired alleles for use in breeding programs is required to generate heat-resistant wheat genotypes.

Wheat consumption is expanding rapidly, with projections of up to 40% growth by 2030. Factors contributing to poorer wheat yield include low-quality seed, wrong broadcasting techniques for sowing, late cultivation, poor soil, uneven fertilizer dosages, inadequate weed eradication, disease, high temperature, and lack of water due to climate change (Ahmed et al., 2020). Due to the rise in consumption, increased wheat production is necessary to ensure global food security. Growing populations, decreased arable land areas, and demand for high nutrition value have posed new challenges for wheat breeders in terms of developing wheat genotypes with specified seed quality; high yield; and drought, heat, and disease resistance (Sukumaran et al., 2018; Gulnaz et al., 2019).

One of the most crucial techniques in wheat breeding is the indirect selection of attributes related to grain production (Gegas et al., 2010). When comparing different spikelets, the grains might differ in grain developmental stages, weight, grain number, height, length, area, width, and sphericity (Ahmed et al., 2018). These phenomena are also seen within individual spikes. The central spikelet in a spike has a more fully developed and heavier grain than spikelets at the basal and top portions of the spike (Boz et al., 2012; Li et al., 2016). The number of spikelets per spike, grains per spikelet, and average grain weight significantly affect grain weight and grains per spike (Guo et al., 2018). While previous studies focused on increasing yield by increasing the number of grains, yield can also be increased by increasing grain size. Grain yield is positively affected by grain size as it increases grain weight, which ultimately increases the wheat yield (Gegas et al., 2010; Boz et al., 2012). Wheat grain yield is not only affected by grain shape and size because large, spherical grains are suitable for milling but shriveled and tiny seeds also reduce milling quality in flour extraction (Li et al., 2016).

Wheat grain has its own set of physical and chemical characteristics. Understanding how wheat grain yield and grain morphological traits are passed down through the generations is essential for developing high-yielding cultivars with superior grain quality (Li et al., 2012). Grain size is a crucial yield component, and the ability to track alleles for bolder, larger grains using precise, gene-based SNP markers should assist in boosting milling yield and increasing the total yield of genotypes. It could also aid in enhancing yield stability (Abdipour et al., 2016).

Smaller grains are more rigid and have worse milling and baking qualities, whereas bigger grains have more endosperm and are heavier. Grain length, rather than breadth or height, is directly linked to grain weight and volume. The quality and yield of wheat flour are determined by grain properties, which are considered during the milling process. These characteristics are tightly linked to various circumstances, the most significant of which is the genetic background (Gegas et al., 2010; Abdipour et al., 2016; Cristina et al., 2016).

Low rates of genetic improvements for wheat yield constitute a genuine danger to global food security, despite increasing human populations and wheat demand (Ray et al., 2013). Thus, identifying, understanding, and incorporating genes that boost wheat production potential across diverse conditions are critical. The polygenic control of grain yield, as well as the influence of the environment, makes this a challenging endeavor. Overall, grain yield can be split into different components to make research more accessible. Total grain weight (TGW) and its physical properties (grain length, breadth, and area) are studied because they are relatively stable and have higher heritability values than total yield (Kuchel et al., 2007). Any additional information on the genetic mechanisms involved in grain size and weight will enhance the efficiency of breeding programs and ensure sustainable production in drought and heat-stress conditions.

The combination of high-density SNP arrays and GWAS (genome-wide association studies) in wheat has been used to identify SNPs associated with a specific trait. Gene prediction and validation are accelerated by the high mapping resolutions of GWAS, which allow researchers to accurately delimit chromosomal areas containing specific loci (Su et al., 2019). Because GWAS can identify whole-genome changes using a variety of panels, it can circumvent the drawbacks of bi-parental populations and save costs and time (Qaseem et al., 2018). A GWAS in bread wheat has been widely utilized to identify essential markers on the A, B, and D genomes separately (Sukumaran et al., 2018). GWAS has developed into a powerful and ubiquitous tool for investigating complex traits (Tadesse et al., 2015). The present study was performed to add knowledge about the genetic basis of drought and heat tolerance in wheat based on grain morphology and yield-related traits.

## Materials and methods

A total of 105 bread wheat genotypes were sown during two growing seasons, 2019–20 and 2020–21, in three different environmental conditions: control, drought, and heat-stress. The genotype names, pedigree records (if available), and origins of the 105 genotypes are listed in Supplementary Table S1. All genotypes were grown in three sets of experiments: control, drought, and heat-stress. These experiments applied a randomized complete block design (RCBD) with three replicates.

In the control experiment, irrigation was applied at three critical stages: first, at tillering (35 days after sowing [DAS]); second, at the booting stage (85 DAS); and third, at the milking stage (112 DAS) (Noorka and Teixeira da Silva 2014). Drought stress was induced at the tillering stage in the drought experiment by skipping the irrigation. For the application of heat stress, the experiment was conducted in a walking tunnel in which one set of the 105 wheat genotypes was seeded. A plastic sheet covered the tunnel during the grain-filling stage to provide high-temperature stress. The temperatures inside and outside the tunnel were recorded daily and were maintained at around 40°C inside the tunnel. In each experiment, all genotypes were seeded in three replicates in a 1-m-long row, with a plant-to-plant spacing of 15 cm and a space between rows of 30 cm. In all environmental conditions (control, drought, and heat-stress), two seeds of each genotype were dibbled per hole, and one healthy wheat seedling was saved following germination by thinning. Fertilizer was applied (NPK 120-90-60 kg/ha).

Standard agronomic practices were adopted as needed in the three environmental conditions. Data for the following characteristics were recorded, and the average values were calculated. Spikes of randomly selected plants were harvested at physiological maturity. The yield and morphological attributes of grain were measured (Ahmed et al., 2018; Gao et al., 2021). A total of 1,000 grains were taken from bulk seeds and weighed using an electric balance (Compax- Cx-600) to determine the thousand-grain weight. The YP was taken by thrashing all spikes from the single plants selected from each replication and then weighing the grain.

The data were analyzed using the analysis of variance (ANOVA) method (Steel and Torrie 1980) in GenStat (v10). The correlations were calculated to evaluate the associations among grain morphology and yield-related traits under all studied environmental conditions. For each variable, the broad sense heritability was determined using the equation given as follows (Sorkheh et al., 2008; Qaseem et al., 2018; Ahmed et al., 2021).

$$H^{2}=\sigma_{g}^{2}/\left(\sigma_{g}^{2}+\left(\frac{\sigma_{g\times e}^{2}}{ny}\right)+\left(\frac{\sigma_{e}^{2}}{ny\times nr}\right.\right)$$

The correlations were calculated in Minitab v16 (Ogunbayo et al., 2005). The significance levels were set to α = 0.01 and α = 0.05, respectively, for ANOVA and correlation coefficients in this study.

### Genotyping of 105 bread wheat genotypes

The seeds from each genotype were sown in germination trays. Fresh leaf samples for DNA isolation were taken from seedlings at 15 days. DNA was isolated as described by Dreisigacker et al. (2013). The extracted DNA (50–100 ng/μl per sample) was genotyped on high-density Illumina 90K Infinium SNP arrays (Wang et al., 2014). The genome-wide locations of SNPs and genetic distance (cM) on each chromosome were determined using the bread wheat genetic map reported by Wang et al. (2014). When evaluating the data, minor alleles, monomorphic SNPs, missing values >20%, and allele frequencies <5% were removed.

### Genome-wide association study

Advanced statistical techniques are used by the Genome Association and Prediction Integrated Tool (GAPIT), including the compressed mixed linear model (CMLM) and CMLM-based genomic prediction and selection (Lipka et al., 2012). This tool was developed as an R package to provide maximum likelihood accuracy and was executed and analyzed in RStudio (Team 2019). The threshold level for significantly associated markers of the studied traits was ≥10^−3^ (log10p) (Sukumaran et al., 2018) after Bonferroni adjustment (*p* = 1/n, n = total numbers of SNPs) applying a correction for a false discovery rate (FDR) <0.05 (Benjamini and Hochberg 1995). Overall, 33,212 of the functional iSelect bead 90K SNP chip analyses visually showed polymorphisms in the examined germplasm and were located on the genetic map (Wang et al., 2014).

## Results

### Phenotypic evaluation

On the examined variables, ANOVA analysis revealed considerable variations in the genotype and environment and their interaction effects (Table 1). Significant genotypic variations (*p <* 0.01) were observed for all examined traits among genotypes identified in the ANOVA. Among environmental effects, significant differences were observed for the studied attributes. The Genotype × Interaction (G × E) interaction between environmental conditions (control, drought, and heat-stress) also showed highly significant differences in the observed attributes (Table 1). Broad sense heritability of the observed traits under consideration was also calculated (Table 1) for both seasons using the values of the variance from ANOVA for each year. High heritability values were observed for grain length, at 76.18% in season 1 and 70.30% in season 2. Grain width and diameter showed heritability values of 70.66% and 69.71%, respectively, in season 1 and 63.07% and 67.07% in season 2. The trait grain roundness showed heritability values of 65.69% and 68.03% in seasons 1 and 2, respectively. Grain circumference and grain surface area showed heritability values of 50.9% and 50.34%, respectively, in season 1 and 56.59% and 53.79% in season 2. The heritability values for thousand-grain weight and grain yield per plant were 67.60% and 64.45%, respectively, in season 1 and 63.22% and 68.18% in season 2.

**TABLE 1:** Heritability broad sense (HBS) and mean sum of squares (MSS) of 105 bread wheat genotypes for the examined traits in both seasons.

Source	Df/	GL		GW		GD		GR		GC		GSA		TGW		GYP	
	Season	S1	S2	S1	S2	S1	S2	S1	S2	S1	S2	S1	S2	S1	S2	S1	S2
REP	2	1005	902	7.22	8.01	5.11	3.01	6.56	7.11	44.51	52.29	8.45	9.34	86.67	95.81	51.33	59.77
GET	104	51.1 **	49.50 **	41.2 **	36.8 **	39.44 **	37.11 **	41.21 **	38.39**	211.22 **	301.88 **	41.87 **	42.45	402.41 **	380.83**	203.34 **	244.98 **
Environment	2	9242 **	9232 **	3998.5 *	42.82	3322.33 *	3644.32 *	611.66 *	490.11 **	618.31 *	501.11	722.01 *	610.22 **	674.49**	614.63 *	501.35 **	399.92 *
GET*Env	208	8.55 **	9.11 **	20.20**	21.01 **	17.44 **	21.44 **	19.88 **	19.28 **	201.22 **	201.12 **	25.98 **	31.33 **	127.88 **	201.56 **	113.66 **	177.88 **
Error	628	4.82	6.11	5.01	6.01	4.99	5.22	6.11	5.2	51.39	61.48	10.36	9.45	55.44	61.86	31.58	32.98
Total	944																
H^2 (%)^		76.19	70.3	70.66	63.07	69.71	67.07	65.69	68.03	50.9	56.59	50.34	53.79	67.6	63.22	64.45	68.18

S1, season 1 (2019–2020); S2, season 2 (2020–2021); REP, replicate; GET, genotypes; Env, environment; DF, degree of freedom; H^2^, heritability; GL, grain length; GW, grain width; GD, grain diameter; GR, grain roundness; GC, grain circumference; GSA, grain surface area; TGW, thousand-grain weight, GYP, grain yield per plant. *significant (*α* = 0.05); **highly significant (*α* = 0.01).

The phenotypic characteristics also showed a wide range of variation. The descriptive statistics of these grain morphology and yield-related traits are presented in Table 2 based on data averaged over the years. The grain length mean values ranged from 5.87 to 10.21 mm, 4.99–9.01 mm, and 4.66–8.88 mm in the control, drought, and heat-stress environments, respectively. The maximum mean values for grain width were 4.18 mm (control), 3.97 mm (drought), and 3.66 mm (heat-stress), while the minimum mean values were 3.09 mm, 2.92 mm, and 2.77 mm, respectively. The variations in grain diameter ranged from 4.30 to 6.16 mm (control), 4.02 to 5.77 mm (drought), and 3.92 to 5.30 mm (heat-stress). The average grain roundness values were 0.58, 0.51, and 0.47 mm in the control, drought, and heat-stress conditions, respectively. Mean grain circumference values were 19.02 mm (control), 18.22 mm (drought), and 17.07 mm (heat stress) among the 105 bread wheat genotypes. The highest mean values for grain surface area were 29.3, 27.78, and 25.02 mm^2^, while the lowest mean values were 15.07, 13.99, and 12.88 mm^2^ in the control, drought, and heat-stress conditions, respectively. The mean thousand-grain weight values ranged from 46.51 to 71.25 g (control), 32.08 to 48.81 g (drought), and 30.08 to 45.03 g (heat). The mean grain yield per plant ranged from 17.15 to 27.18 g (control), 16.05 to 21.01 g (drought), and 15.34 to 20.2 g (heat stress).

**TABLE 2:** Descriptive statistics for the eight-grain seed morphology and yield-related traits evaluated in three different environments based on averages over the years (2019–2021).

Trait (Unit)	Environment	Range	Minimum	Maximum	Mean	SD	CV%
Grain length (mm)	N	5.87–10.21	5.87	10.21	6.98	0.51	7.31
	D	4.99–9.01	4.99	9.01	6.87	0.49	7.13
	H	4.66–8.88	4.66	8.88	6.45	0.52	8.06
Grain width (mm)	N	3.09–4.18	3.09	4.18	3.98	0.18	4.52
	D	2.92–3.97	2.92	3.97	3.63	0.19	5.23
	H	2.77–3.66	2.77	3.66	3.35	0.17	5.07
Grain diameter (mm)	N	4.30–6.16	4.3	6.16	5.01	0.23	4.59
	D	4.02–5.77	4.02	5.77	4.91	0.21	4.28
	H	3.92–5.30	3.92	5.3	4.27	0.25	5.85
Grain roundness (mm)	N	0.44–0.66	0.44	0.66	0.58	0.04	6.90
	D	0.37–0.61	0.37	0.61	0.51	0.03	5.88
	H	0.33–0.58	0.33	0.58	0.47	0.03	6.38
Grain circumference (mm)	N	16.01–25.22	16.01	25.22	19.02	0.75	3.94
	D	14.88–23.18	14.88	23.18	18.22	0.75	4.12
	H	13.96–21.21	13.96	21.21	17.07	0.73	4.28
Grain surface area (mm^2^)	N	15.07–29.30	15.07	29.3	20.34	1.51	7.42
	D	13.99–27.78	13.99	27.78	19.81	1.52	7.67
	H	12.88–25.02	12.88	25.02	18.24	1.48	8.11
Thousand grain weight (g)	N	46.51–71.25	46.51	71.25	54.03	2.89	5.35
	D	32.08–48.81	32.08	48.81	41.52	2.82	6.79
	H	30.08–45.03	30.08	45.03	39.01	2.81	7.20
Grain yield per plant (g)	N	17.15–27.18	17.15	27.18	19.73	1.54	7.81
	D	16.05–21.01	16.05	21.01	17	1.47	8.65
	H	15.34–20.2	15.34	20.2	16.11	1.49	9.25

Based on the average data per year, Pearson’s correlation coefficients were determined for all attributes in the control, drought, and heat-stress conditions (Table3). A significant positive correlation was observed among GD and GSA (0.95), followed by between GD and GC (0.94), in the heat-stress condition. A significantly positive association was observed among GL, GD, GC, and GSA traits under the control, heat, and drought conditions. However, GL had a slight positive association with GW, TGW, and GYP under all studied environmental conditions. The most negative but significant correlation (−0.49) was observed between GL and GR under the heat-stress condition. Yield-related traits like TGW and GYP were significantly correlated with grain morphology-related traits like GW, GC, and GSA under all examined environmental conditions.

**TABLE 3:** Pearson’s correlation results of grain physical attributes in spring wheat genotypes based on data averaged over the years (2019–2021).

Traits	Environment	GL	GW	GD	GR	GC	GSA	TGW
GW	Control	0.21						
	Drought	0.19						
	Heat	0.17						
GD	Control	0.74**	0.66**					
	Drought	0.69**	0.62**					
	Heat	0.70**	0.69**					
GR	Control	−0.48**	0.52**	0.75**				
	Drought	−0.49**	0.56**	0.79**				
	Heat	−0.46**	0.59**	0.81**				
GC	Control	0.77**	0.39*	0.91**	−0.31*			
	Drought	0.81**	0.37*	0.93**	−0.25			
	Heat	0.72**	0.35*	0.94**	−0.33			
GSA	Control	0.74**	0.65**	0.93**	0.26	0.88**		
	Drought	0.71**	0.66*	0.90**	0.18	0.85**		
	Heat	0.69**	0.71**	0.95**	0.19	0.89**		
TGW	Control	0.28	0.66**	0.51**	0.46*	0.58**	0.47*	
	Drought	0.31*	0.71**	0.53**	0.48*	0.61**	0.34*	
	Heat	0.29	0.68**	0.49**	0.47*	0.63*	0.44*	
GYP	Control	0.13	0.64**	0.57**	0.39*	0.45*	0.51**	0.88*
	Drought	0.18	0.77**	0.61**	0.37*	0.46*	0.55**	0.82*
	Heat	0.15	0.88**	0.62**	0.41**	0.49*	0.59**	0.78*

GL, grain length; GW, grain width; GD, grain diameter; GR, grain roundness; GC, grain circumference; GSA, grain surface area; TGW, thousand-grain weight; GYP, grain yield per plant.

### Marker-trait associations

**FIGURE 1:**
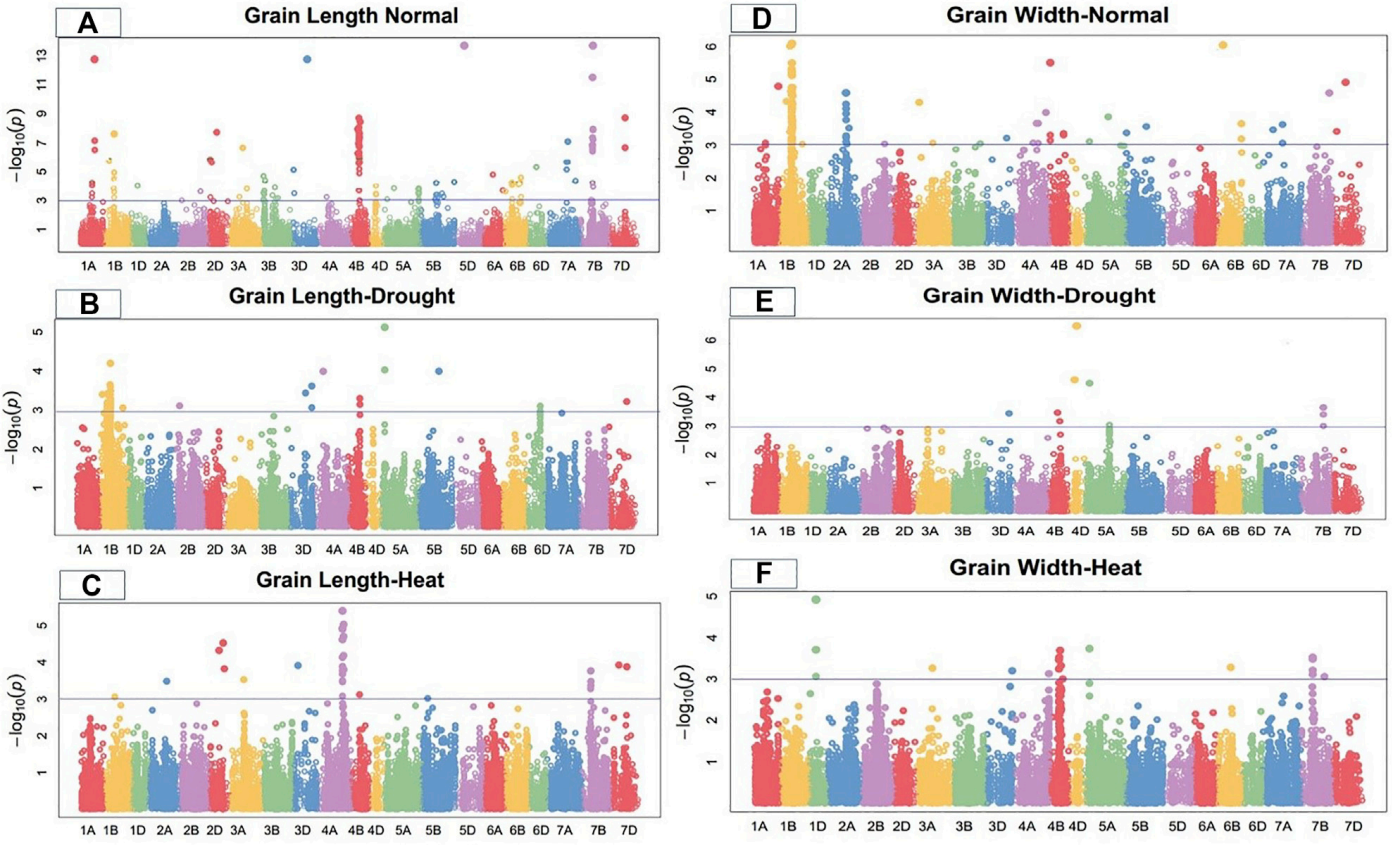
Manhattan plot of grain length and width under control, drought, and heat-stress conditions.

**FIGURE 2:**
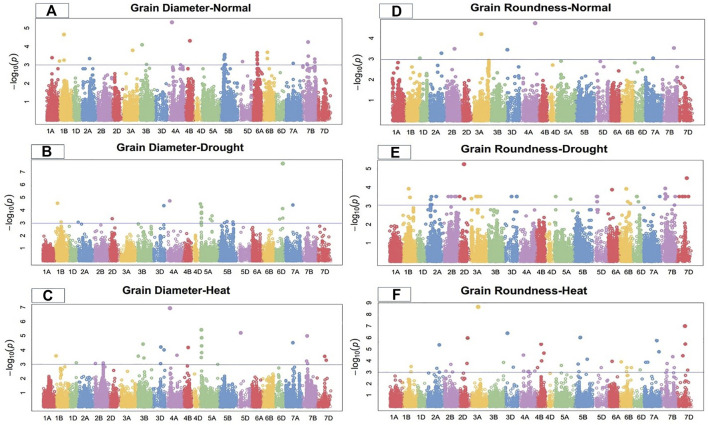
Manhattan plot of grain diameter and roundness under control, drought, and heat-stress conditions.

**FIGURE 3:**
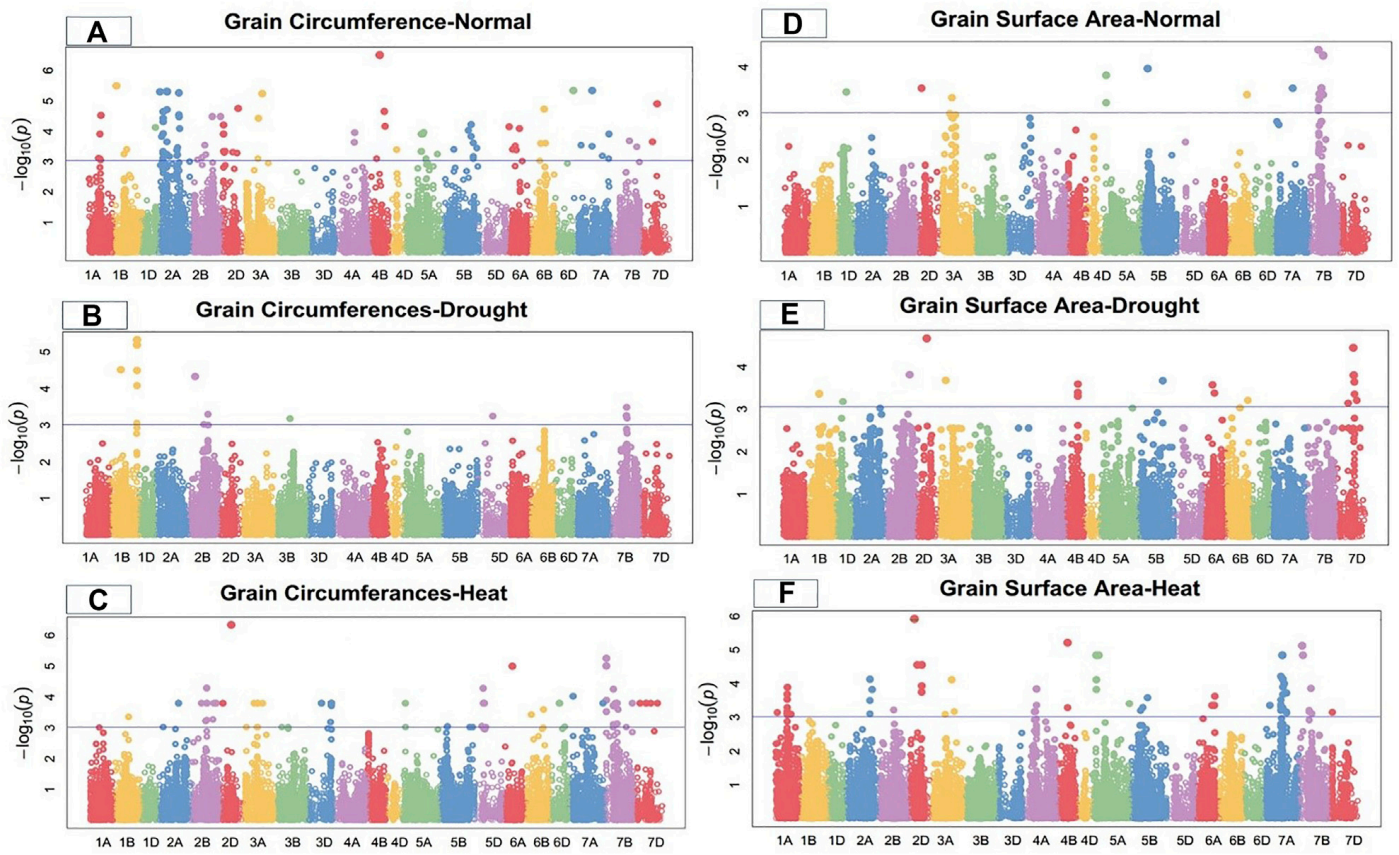
Manhattan plot of grain circumference and surface area under control, drought, and heat-stress conditions.

**FIGURE 4:**
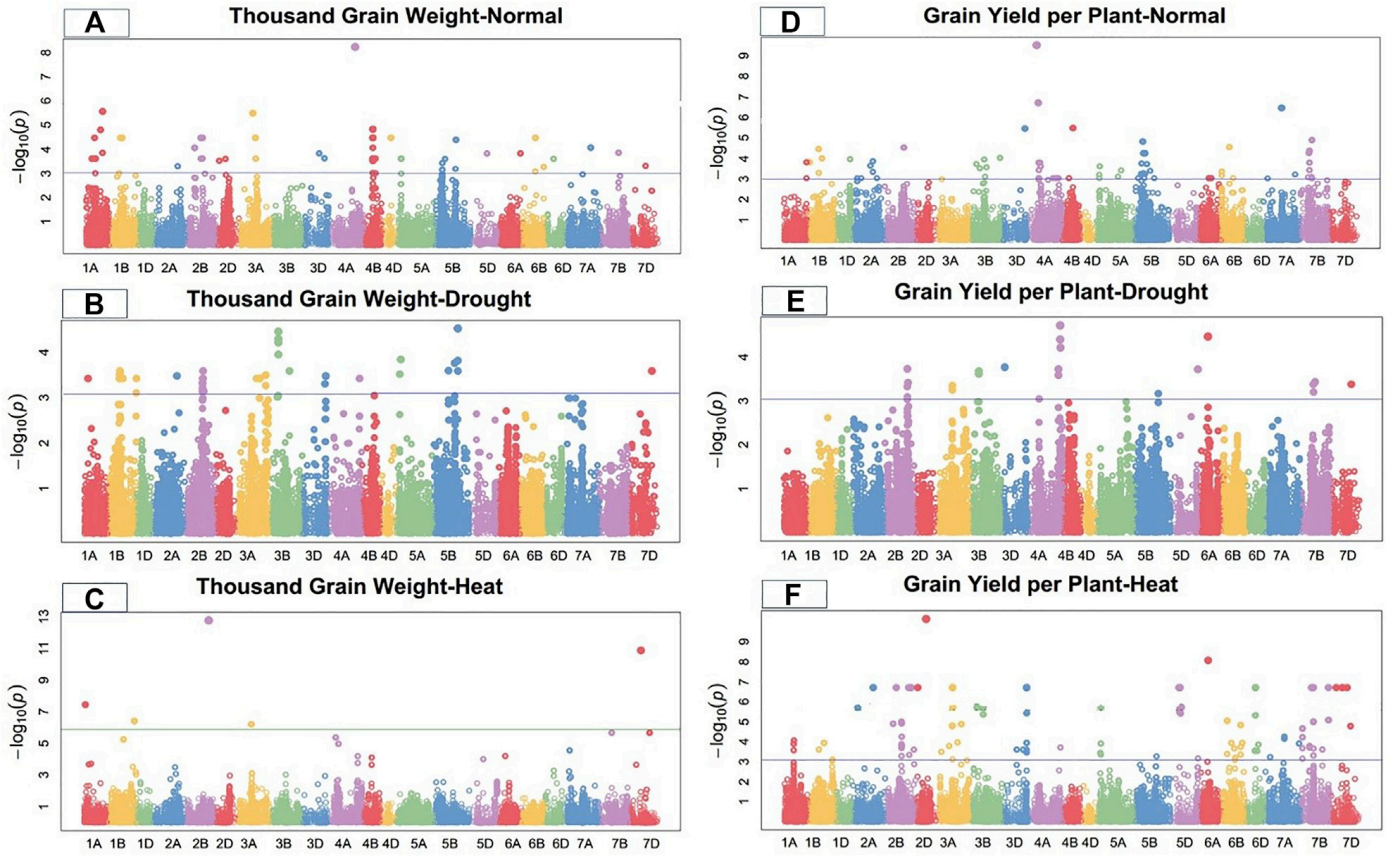
Manhattan plot of thousand-grain length and grain yield per plant under control, drought, and heat-stress conditions.

Marker-trait associations (MTAs) for grain morphology and yield traits under control, drought, and heat-stress conditions were examined and visualized using Manhattan plots (Figures 1–4). These figures show the site of significantly associated SNPs according to p values -log10(p), linked to the studied traits in all studied environmental conditions. In this study, a total of 541 SNPs showed significant associations; of these, 195, 179, and 167 significant MTAs were observed in the control (Supplementary Table S2), drought (Supplementary Table S3), and heat‐stressed (Supplementary Table S4) conditions, respectively, at the–log 10 (*p ≤* 10^−3^) threshold level using FDR *≤*0.05 correction in the studied bread wheat genotypes.

### Grain length

Under control conditions, 50 SNPs located on chromosomes 1A, 1B, 2D, 3D, 4B, 5D, 7A, 7B, and 7D were significantly linked to grain length in the current GWAS study (Figure 1). These loci ranged from 20.78 to 32.71% of the total phenotypic variation for this attribute. Under control conditions, marker *Kukri rep c71356 236* at 244.31 cM on chromosome 7B showed the highest trait phenotypic variation (32.71%). In comparison, marker *Tdurum contig76677 1142* on chromosome 4B at 176.21 cM showed the lowest phenotypic variation in grain length (20.78%) (Supplementary Table S2).

On chromosomes 1B, 3D, 4A, 4B, 5A, 5B, and 7D, 26 SNPs were highly related to grain length under drought conditions (Figure 1). The total phenotypic variation of these SNPs ranged from 16.08% to 26.62% (Table 5). Under drought conditions, marker *Kukri_c45439_457* had the highest phenotypic variation (PV) (26.62%) at 78.15 cM on chromosome 5A. In comparison, marker *Tdurum_contig87227_108* showed the lowest PV (16.09%) at 201.25 cM on chromosome 1B. Significantly associated MTAs were dispersed on all wheat genomes, including three from A-genome, 20 from B-genome, and three from D-genome in the drought condition (Supplementary Table S3). Under the heat-stress condition, grain length was significantly linked with 18 SNPs. Seven significant MTAs associated with GL were identified on chromosomes 4A; three on 2D; two each on 4B and 7D; and one on each 1B, 2A, 3A, and 3D (Figure 1). These eighteen GL-related SNPs accounted for 12.33%–25.36% of the PV in this attribute (Supplementary Table S4). Marker *Excalibur_c22896_149* at position 475.83 cM on chromosome 4A showed the highest PV (25.36%).

### Grain width

Under the control condition, grain width was highly associated with 25 SNPs. Seventeen SNPs were located on chromosome 1B; three on 2A; and one on each of 1A, 4B, 6B, 7B, and 7D (Figure 1). These grain-width-related SNPs accounted for 21.64%–30.77% of the total PV in grain width (Supplementary Table S2). Marker *BobWhite_c35520_397* at 230.29 cM on chromosome 1B showed the highest PV (30.77%), while marker *RAC875_rep_c111001_187* at 244.91 cM on the same chromosome showed the lowest PV (21.64%). MTAs for grain width were distributed across all wheat genomes, with four SNPs on the A-genome, 20 on the B-genome, and one on the D-genome under the control condition.

Under the drought condition, 12 SNPs were significantly linked with grain width, with four SNPs on chromosome 7B, three on 4D, two on 4B, two on 5A, and one on 3D (Figure 1). These SNPs accounted for 12.17%–31.92% of the total PV in grain width under the drought condition. The significantly associated SNPs were distributed among all wheat genomes, with two from the A-genome, six from the B genome, and four from the D genome. Marker *BobWhite_c43880_73* at 159.35 cM on chromosome 4D showed the highest phenotypic variation (31.92%), while marker *Excalibur_c7338_563* at 427.7 cM on 7B showed (Supplementary Table S3) the lowest PV (12.17%) for this trait under the drought condition.

In the heat-stress conditions, 16 SNPs were significantly positively associated with grain width, including four SNPs each on chromosomes 4B and 7B; three SNPs on 1D; and one each on chromosomes 3A, 3D, 4A, 5A, and 6B (Figure 1). These SNPs showed PV in grain width ranging from 12.17% to 22.48% under the heat-stress condition. Marker *BS00022027_51* at 108.87 cM on chromosome 1D showed the highest PV (22.48%) (Supplementary Table S4).

### Grain diameter

Under the control condition, grain diameter was highly associated with 38 SNPs, 12 of which were located on chromosome 5B, followed by six on 6A, five on 7B, three on 1B, two each on 3B and 6B, and one each on 1A, 2A, 3A, 4B, 5D, and 7A (Figure 2). These SNPs comprised 15.42% to 28.03% of the total PV in grain diameter. The MTAs for this trait were distributed across three genomes, with 12 SNPs on the A-genome, 25 on the B-genome, and one on the D-genome. Marker *Tdurum_contig48049_705* located at 160.42 cM on chromosome 4A showed the highest PV (28.03%), while marker *Tdurum_contig93156_239* at 497.16 cM on the same chromosome showed the lowest PV (15.42%) (Supplementary Table S2).

Under the drought condition, 21 SNPs were significantly associated with grain diameter. Eight SNPs were located on chromosome 5A, five on 6D, two each on chromosomes 1B and 5B, and one each on chromosomes 2A, 2D, 3D, 4A, and 7A (Figure 2). Under the drought condition, these significant SNPs showed total PV in grain diameter ranging from 12.27% to 21.48%. The MTAs for grain diameter were distributed across all wheat genomes, with 11 SNPs on the A-genome, four on the B-genome, and six on the D-genome (Supplementary Table S3). Marker *Tdurum_contig48049_705* at 160.42 cM on chromosome 4A showed the highest PV (21.48%), while marker *BS00067501_51* at 222.57 cM on chromosome 5B showed the lowest PV (12.27%).

Under the heat-stress condition, grain diameter was positively correlated with 20 SNPs. Three SNPs each were located on chromosomes 3D and 7B; two each on chromosomes 2B, 3B 5A, and 7D; and one each on chromosomes 1B, 1D, 4A, 4B, 5D, and 7A (Figure 2). These SNPs comprised 17.49% to 29.88% of the total PV for grain diameter under this condition. Significant MTAs were distributed across all wheat genomes, including four SNPs from the A-genome, nine from B-genome, and seven from the D-genome. Marker *Tdurum_contig14863_885* at 67.48 cM on chromosome 5A showed the highest PV (29.88%), while marker *Kukri_c23474_718* 283.69 cM on chromosome 3D showed the lowest PV (17.49%) (Supplementary Table S4).

### Grain roundness

Under the control condition, a total of eight significant SNPs were observed for grain roundness. One SNP each was located on chromosomes 1D, 2A, 2B, 3A, 3D, 4A, 7A, and 7B (Figure 2). The total PV in grain roundness comprised by these SNPs ranged from 12.28% to 21.32%. Marker *Tdurum_contig100702_265* at 542.67 cM on chromosome 4A showed the highest PV (21.32%), while marker *Kukri_c13134_132* at 5.63 cM on chromosome 1D showed the least PV (12.28%) (Supplementary Table S2). The MTAs for grain roundness attributes were distributed across three wheat genomes, four on the A-genome and two each on the B and D-genomes.

Under the drought condition, 37 SNPs were significantly associated with grain roundness. Six were located on chromosome 2A; five each on 3D, 5D, and 6D; four on 7B, two each on 2B, 5A, 6B, and 7D; and the remaining on 1B, 2D, 6A, and 7A (Figure 2). These SNPs had a total PV in grain roundness under the drought condition ranging from 13.27% to 25.27%. The MTAs for this trait were distributed across three wheat genomes, with 10 SNPs on the A-genome, eight on the B-genome, and 18 on the D-genome. Marker *D_contig57523_172* at 183.76 cM on chromosome 2D showed the highest variation (25.27%), while marker *Kukri_c31508_91* at 176.43 cM on chromosome 2A showed the least PV (13.27%) for grain roundness under the drought-stress condition (Supplementary Table S3).

Under the heat-stress condition, 25 MTAs were significantly correlated with grain roundness. These MTAs were located on chromosomes 1B, 2A, 2B, 2D, 3B, 3D, 4A, 4B, 5B, 5D, 6D, 7A, 7B, and 7D (Figure 2) and accounted for PV ranging from 12.18% to 35.01%. Marker *D_GB5Y7FA02JIMB5_49* at 290.6 cM on chromosome 7D showed the highest variation (35.01%), while marker *Ex_c10068_1509* at 479.11 cM on chromosome 2A showed the lowest PV (12.18%) for grain roundness under the heat-stress condition. The MTAs were distributed on all wheat genomes, with seven on the A-genome and nine each on the B- and D-genomes (Supplementary Table S4).

### Grain circumference

Under the control condition, 15 SNPs on chromosomes 1B, 2A, 2D, 3A, 4B, 6B, 6D, 7A, and 7D were highly associated with grain circumference (Figure 3). These SNPs accounted for total PV in grain circumference ranging from 21.64% to 32.58%. The MTAs were distributed across all wheat genomes, with four SNPs on the A-genome and two each on the B- and D-genomes. Marker *Tdurum_contig82633_313* at 176.21 cM on chromosome 4B showed the highest PV (32.58%), while marker *BS00067342_51* at 86.88 cM on chromosome 2A showed the lowest PV (21.64%) (Supplementary Table S2).

Under the drought condition, 13 SNPs were associated with grain circumferences. Four SNPs each were located on chromosomes 1B and 7B, three on 2B, and one each on 3B and 5D (Figure 3). These SNPs comprised 12.25%–24.97% of the PV for grain circumference. Marker *Excalibur_c39284_949* at 510.03 cM on chromosome 1B showed the highest PV (27.55%), while marker *Ex_c13213_2992* at 371.86 cM on chromosome 2B showed the lowest PV (16.75%) (Supplementary Table S3) for grain circumference under the drought condition.

Under the heat-stress condition, the grain circumference was highly correlated with 21 MTAs, five of which were located on chromosome 7B, three on 2B, two each on 5D and 6D, and one each on chromosomes 1A, 2D, 3A, 3D, 5B, 6A, 6B, 7A, and 7D (Figure 3). The PV ranged from 12.46% to 31.12%. Significant MTAs for GC were distributed on all wheat genomes, with four SNPs from the A-genome, ten from the B-genome, and seven from the D-genome (Supplementary Table S4). Marker *D_contig57523_172* at 183.76 cM on chromosome 2D showed high PV (31.12%) under the heat-stress condition.

### Grain surface area

Under the control condition, grain surface area was highly associated with 18 SNPs, including 15 on chromosome 7B and one each on chromosomes 5A, 5B, and 7A (Figure 3). The total PV of these GSA-related SNPs ranged from 14.96% to 19.42% (Supplementary Table S2). Marker *BS00035630_51* at 172.1 cM on chromosome 7B showed the highest PV variation (19.42%), while marker *Excalibur_c22340_449* at 398.79 cM on chromosome 7A showed the lowest PV (14.96%). The MTAs for GCA were distributed across two wheat genomes, with 16 SNPs on the B-genome and two on the B-genome under the control condition.

Under the drought condition, 22 SNPs were significantly linked to grain surface area, including seven SNPs on chromosome 7D, three on 4B, two each on 6A and 6B, and one each on 1B, 1D, 2A, 2B, 2D, 3A, 5A, and 5B (Figure 3). These SNPs showed total PV in GSA ranging from 14.67% to 23.19% under the drought condition. Marker *D_contig57523_172* at 183.76 cM on chromosome 2D showed the highest PV (23.19%), while marker *Tdurum_contig64286_268* at 517.73 cM on chromosome 2A showed the lowest PV (14.67%) for grain surface area under the drought condition (Supplementary Table S3).

Under the heat-stress condition, 22 SNPs were significantly associated with grain surface area, with PV ranging from 24.85% to 34.88%. Four SNPs each were detected on chromosomes 1A, 7A, and 7B; three on 5A; two on 2A; and one each on 1B, 3B, and 4B (Figure 3). Marker *Excalibur_rep_c69263_462* at 182.55 cM on chromosome 4B showed the highest PV (34.88%), while marker *BS00100120_51* at 260.38 cM on chromosome 1A showed the lowest PV (24.85%) for grain surface area under the heat-stress condition (Supplementary Table S4).

### Thousand-grain weight

Under the control condition, thousand-grain weight was significantly linked with 22 SNPs. Ten significant MTAs associated with TGW were detected on chromosome 4B; three on 2B, two each on 1A, 1B, and 3A; and one each on 4D, 5B, and 6B (Figure 4). These 22 TGW-related SNPs showed a total PV in thousand-grain weight ranging from 19.52%–26.26% (Supplementary Table S2). Marker *CAP11_c2285_104* at 357.33 cM on chromosome 1A showed the highest PV (26.26%), while marker *Tdurum_contig50731_961* at 400.89 cM on chromosome 5B showed the minimum PV (19.52%). The significant MTAs for thousand-grain weight were distributed on all wheat genomes, with four SNPs on the A-genome, 17 on the B-genome, and one on the D-genome.

Under the drought-stress condition, 28 SNPs were positively significantly associated with TGW, including six SNPs on chromosome 1B, four each on chromosomes 2B and 3B, three each on each chromosomes 3A and 5B, and one each on chromosomes 1A, 2A, 3D, 4A, 4B, and 7D (Figure4). These markers showed a total PV ranging from 12.21% to 20.23% under the drought condition. Marker *Tdurum_contig58293_437* at 461.54 cM on chromosome 5B showed the highest PV (20.23%), while marker *Ku_c9596_1649* at 113.62 cM on chromosome 3B showed the lowest PV (12.21%) (Supplementary Table S3). The significant MTAs for thousand-grain weight were distributed on all wheat genomes, with eight SNPs in the A-genome, 18 on the B-genome, and two on the D-genome.

Under the heat-stress condition, 24 SNPs on chromosomes 1A, 1B, 2A, 3A, 3B, 4A, 4B, 5D, 6A, 6D, 7A, 7B, and 7D were highly associated with thousand-grain weight (Figure 4). These SNPs showed total PV ranging from 12.30% to 29.88%. Marker *Tdurum_contig44851_9272* at 513.23 cM on chromosome 1B showed the highest PV (29.887%), while marker *Excalibur_c19552_319* at 278.84 cM on chromosome 3B showed the lowest PV (12.30%) for thousand-grain weight under the heat-stress condition. The significant MTAs for TGW were dispersed on all three genomes, with 12 SNPs on the A-genome, eight on the B-genome, and four on the D-genome (Supplementary Table S4).

### Grain yield per plant

Under the control condition, grain yield per plant was significantly linked with 19 SNPs on chromosomes 1B, 2B, 3B, 3D, 4A, 4B, 5B, 6B, 7A, and 7B (Figure 4). These sixteen GYP-related markers showed total PV ranging from 18.35% to 36.38% (Supplementary Table S2). Marker *Tdurum_contig48231_1233* at 191.56 cM on chromosome 4A showed the highest PV (34.04%), while marker *BobWhite_c34267_459* at 273.29 cM on chromosome 1B showed the lowest PV (18.41%). The significant MTAs for this grain per yield were distributed on all wheat genomes, with two SNPs of the A-genome, 16 on the B-genome, and one on the D-genome under the control condition.

Under the drought condition, 20 SNPs were significantly linked with GYP, with five SNPs each on chromosomes 2B and 4A; two SNPs each on 3A and 7B; and one SNP each on 3B, 3D, 5B, 5D, 6A, and 7D (Figure 4). Marker *RAC875_s119811_122* at 597.71 cM on chromosome 4A showed the highest PV (21.36%), while marker *Tdurum_contig49532_53* at 429.47 cM on chromosome 2B showed the lowest PV (12.34%) under the drought condition. The significant MTAs for grain yield per plant were distributed on all wheat genomes, with eight SNPs on the A-genome, nine on the B-genome, and three on the D-genome. These SNPs showed a total PV ranging from 12.34% to 21.36% under the drought condition (Supplementary Table S3).

Under the heat-stress condition, grain yield per plant was significantly linked to 21 SNPs. Seven MTAs were detected on chromosome 7B, five on 2B, three on 6B, two on 6B, and one on each 1A, 1B, 3A, and 4A (Figure 4). Marker *D-contig24171-152* at 191.27 cM on chromosome 6D showed the highest PV (28.74%), while marker *Excalibur_c58468_162* at 418.37 cM on chromosome 7B showed the lowest PV (20.53%) for grain yield per plant under the heat-stress conditions. These 21 GYP-related SNPs showed a total PV ranging from 20.53% to 28.74% (Supplementary Table S4).

### Trait-wise and genome-wide marker-trait associations

The highest numbers of MTAs were identified for GL (50) followed by GD (38), GW (25), TGW (22), GSA (18), GYP (19), GC (15), and GR (8) under the control condition (Table 4). Under the drought-stress condition, the highest number of MTAs was identified in GR (37), followed by TGW (28), GL (26), GSA (22), GD (21), GYP (20), GC (13), and GW (12). In the heat-stress condition, the highest number of MTAs was observed in GR (25), followed by TGW (24), GSA (22), GYP (21), GC (21), GL (18), GW (16), and GD (20). The highest number of MTAs under the control condition was identified on chromosome 4B (45), followed by 7B (34), 1B (29), 5B (21), and 2 A (11), while the D-genome showed the lowest number of MTAs (13). However, the B-genome showed the highest number (143), with the A-genome in between (39). Under the drought condition, the highest number of MTAs was observed on chromosomes 1B (31), followed by 5A (17), 2B (15), 7B (14), 7D (12), and 3D (11). The D- and A-genomes showed the lowest numbers of MTAs (46 and 47, respectively), while the B-genome had the highest number (86) (Table 4). Under the heat-stress condition, the highest number of MTAs was identified on chromosome 7B (25), followed by 4A (18), 2B (12), 7D (11), and 4B (11). The D-genome showed the lowest number of MTAs (40), while the B-genome showed the highest number (70), followed by the A-genome (57) under this condition.

**TABLE 4:** Significant MTAs reported in this study.

Significant MTAs							
Genome	Genome-wise and chromosome-wise			Traits-wise			
	Control	Drought	Heat Stress	Traits	Control	Drought	Heat stress
A Genome	Total 39 MTAs (1A = 6.2A = 11,3A = 5, 4A = 4, 5A = 1, 6A = 6, 7A = 6)	Total 47 MTAs (1A = 1, 2A = 9, 3A = 6, 4A = 8, 5A = 17, 6A = 4, 7A = 2)	Total 57 MTAs (1A = 8, 2A = 8, 3A = 5, 4A = 18, 5A = 6, 6A = 3, 7A = 9)	GL	50	26	18
				GW	25	12	16
				GD	38	21	20
B Genome	Total 143 MTAs (1B = 29, 2B = 5, 3B = 3, 4B = 45, 5B = 21, 6B = 6, 7B = 34)	Total 86 MTAs (1B = 31, 2B = 15, 3B = 6, 4B = 8, 5B = 8, 6B = 4, 7B = 14)	Total 70 MTAs (1B = 9, 2B = 12, 3B = 4, 4B = 11, 5B = 4, 6B = 5, 7B = 25)	GR	8	37	25
				GC	15	13	21
				GSA	18	22	22
D Genome	Total 13 MTAs (1D = 1, 2D = 2, 3D = 2, 4D = 1, 5D = 3, 6D = 1, 7D = 4)	Total 46 MTAs (1D = 1, 2D = 3, 3D = 11, 4D = 3, 5D = 7, 6D = 9, 7D = 12)	Total 40 MTAs (1D = 4, 2D = 6, 3D = 8, 5D = 5, 6D = 6, 7D = 11)	TGW	22	28	24
				GYP	19	20	21
				Total	195	179	167

In the A-genome, markers *Tdurum_contig48231_1233*, *Kukri_c45439_457*, and *Ku_c10135_987* at 191.56 cM, 78.15 cM, and 78.15 cM on chromosomes 4A, 5A, and 5A were significantly associated with GYP, GL, and GS under the control, drought, and heat-stress conditions, with the highest PVs of 24.04%, 26.62%, and 33.05%, respectively. The lowest PV (12.30%, 12.37%, and 12.18%) was observed for markers *D_GDEEGVY01CQJ66_272*, *Kukri_c865_59*, and *Ex_c10068_1509* at 375.14 cM, 463.65 cM and 479.11 cM on chromosomes 7A, 5A, and 2A in the A-genome, which were associated with GR, GW, and GR under the control, drought, and heat-stress conditions, respectively. Markers *RFL_Contig1445_1192*, *Excalibur_c7338_563*, and *Excalibur_rep_c103202_402* on the B-genome on chromosomes 2B (343.22 cM), 7B (427.7 cM) and 4B (243.79 cM) showed the lowest PV (14.61%, 12.17%, and 12.17%%) for GR, GW, and GW under the control, drought, and heat-stress conditions, respectively. In the B-genome, markers *Kukri_rep_c71356_236, Excalibur_c39284_949,* and *Excalibur_rep_c69263_462* at 244.31cM and 510.03 cM and 182.55 cM on chromosome 7B, 1B and 4B showed the highest PV (32.71%, 24.97%, and 34.88%) and were significantly associated with GL, GC, and GS under the control, drought, and heat-stress conditions, respectively. Markers *RFL_Contig2949_500, BobWhite_c43880_73*, and *D_GB5Y7FA02JIMB5_49* on chromosomes 5D (194.19 cM), 4D (159.35 cM), and 7D (290.6 cM) on the D-genome were associated with GL, GW, and GR and showed the highest PV (32.39%, 31.92%, and 35.01%) under the control, drought, and heat-stress conditions, respectively. The lowest PV (12.28%, 13.52%, and 12.47%) was observed for markers *Kukri_c13134_132*, *wsnp_RFL_Contig2996_2877869*, and *CAP7_rep_c9997_155* at 5.63cM, 277.56cM and 108.87 cM on chromosomes 1D, 5D, and 1D on the D-genome and were associated with GR, GC, and GW under the control, drought, and heat-stress conditions, respectively.

Under the control and drought conditions, grain morphology and yield attributes showed pleiotropic loci as*BS00010616_51* and *BS00010868_51* at 186.24 cM and 35.47 cM on chromosomes 7B and 1B, respectively (Table 5). Under the control and heat-stress conditions, the studied traits were influenced by pleiotropic loci *BS00010868_51*, *Kukri_c11154_1723*, and *Ex_c10068_1509* at 35.47 cM, 126.02 cM, and 479.11 cM on chromosomes 1B, 5B, and 2A respectively. The pleiotropic loci *Kukri_rep_c111174_132* and *Kukri_c27958_334* at 546.25 cM and 153.27 cM on chromosomes 1B and 6A were linked to grain morphology-related traits under the drought and heat-stress conditions, respectively (Supplementary Table S5). Only one stable and consistent locus (*Excalibur_c20796_395*) at 372.34 cM on chromosome 7A was also linked to grain morphology and yield-related attributes in all three conditions (Table 5).

**TABLE 5:** Pleiotropic loci and stable MTAs across the study environment in 105 bread wheat genotypes.

Pleiotropic loci						
Locus name	Location	Position	Trait	Control	Drought	Heat
Durum_contig44261_127	7B	195.74	GD, GYP	✓	×	×
Excalibur_c20796_395	7A	372.34	GD, GYP	✓	×	×
Excalibur_c41898_218	5B	168.1	GD, GYP	✓	×	×
Excalibur_c766_771	6B	148.84	GD, GYP	✓	×	×
RAC875_c30829_1711	5B	179.66	GD, GYP	✓	×	×
Tdurum_contig13784_824	5B	168.1	GD, GYP	✓	×	×
wsnp_Ex_c2727_5053747	5B	212.2	GD, GYP	✓	×	×
wsnp_RFL_Contig1570_778491	5B	212.38	GD, GYP	✓	×	×
wsnp_RFL_Contig2504_2093982	5B	179.66	GD, GYP	✓	×	×
BS00023035_51	4B	238.43	GSA, TGW	×	✓	×
IACX419	6B	237.57	GR, GSA	×	✓	×
Kukri_c45439_457	5A	78.15	GL, GD	×	✓	×
Tdurum_contig97656_120	3B	136.78	TGW, GYP	×	✓	×
BS00093856_51	3D	72.19	GL, GR	×	×	✓
Ex_c10574_1027	2D	275.13	GL, GR	×	×	✓
Excalibur_c25991_184	7B	169.41	GC, GYP	×	×	✓
GENE-4937_537	2D	298.38	GL, GR	×	×	✓
Stable MTAs						
BS00010616_51	7B	186.24	GYP, GD	✓	×	✓
BS00010868_51	1B	35.47	GD, GYP	✓	×	✓
D_contig22507_191	6D	191.27	GR, GC	×	✓	✓
D_contig24171_152	6D	191.27	GR, GYP	×	✓	✓
D_contig57523_172	2D	183.76	GR, GS, GC	×	✓	✓
D_GCE8AKX01CTXDI_46	6D	191.27	GR, GC	×	✓	✓
Ex_c10068_1509	2A	479.11	GR	✓	×	✓
Excalibur_c20796_395	7A	372.34	GD, GYP	✓	✓	✓
Excalibur_c25991_184	7B	169.41	GR, GC	×	✓	✓
GENE-4403_405	7B	244.31	GYP, GSA	✓	×	✓
GENE-4937_537	2D	298.38	GSA, GL	✓	×	✓
Kukri_c11154_1723	5B	126.02	GSA, GL	✓	×	✓
Kukri_c27958_334	6A	153.27	GSA, GC	×	✓	✓
Kukri_rep_c111174_132	1B	546.25	TGW, GYP	×	✓	✓
Tdurum_contig46313_394	4B	211.4	GYP, GD	✓	×	✓
Tdurum_contig48049_705	4A	160.42	GD, GL	✓	✓	×

## Discussion

### Phenotypic evaluations

Increased grain width contributes to increased grain weight, according to a significant positive association between grain width and diameter and thousand-grain weight. In the current study, however, grain width significantly influenced grain weight more than grain length. Previous studies have reported moderate to high associations between grain weight, grain surface area, and grain circumference (Rasheed et al., 2014). Longer and broader grains can acquire more starch and show greater grain weight; the correlations between these traits lead to a causal link between grain size and weight and, ultimately, increased grain yield per plant. The correlation coefficient between grain size measurements and grain weight is positive and significant (Rasheed et al., 2014). The results of the present study showed a positive association of grain length and width with thousand-grain weight. Russo et al. (2014) also reported a significant positive correlation between grain roundness and grain length, consistent with the finding in the current study. Grain weight is also significantly correlated with grain size and shape (grain length and grain width) (Gegas et al., 2010). Grain size and shape are essential traits in wheat domestication and breeding since they are linked to yield and milling quality. Compared to ancient wheat species, which have more variety in grain size and form, modern wheat cultivars have more comprehensive and shorter grains (Yan et al., 2017). Larger grains may have a positive impact on seedling vigor and enhance output. According to geometrical models, changes in grain size and form might result in up to a 5% increase in flour production. In the present study, the traits with the highest heritability were phenotypic, the most stable yield components. They may be used as independent descriptors in breeding programs to boost grain production. These findings will aid wheat breeders in developing high-yielding genotypes with improved grain architecture to improve bread wheat milling and baking quality.

### Marker-trait associations for grain physical and yield attributes in all studied conditions

The genome-wide analysis has identified several loci linked to grain shape across distinct chromosomal regions in different environmental conditions (Su et al., 2019). This diverse panel was used for other wheat traits but has never been utilized for grain morphology traits using GWAS. This study examined 33,212 high-density SNPs from the 90K Illumina iSelect SNP array (Wang et al., 2014) to detect those associated with grain morphology and yield-related traits. Marker-trait associations (MTAs) were investigated for the examined traits in control, drought, and heat-stressed conditions. We discovered critical genomic areas containing specific vital genes linked to these traits. Grain size is primarily determined by grain weight and area, while grain shape refers to the percentage of the grain’s primary growth axes (Gegas et al., 2010).

Wheat grain size and shape are positively connected with TGW and influence flour yield, end-use quality, and market price (Abdipour et al., 2016). Grain size and shape significantly impact grain weight and is also a major breeding goal due to market and industry demands. Many QTLs influencing grain size and shape have been reported in hexaploid wheat (Simmonds et al., 2016).

Grain width was related to more SNPs than the other variables. Wang et al. (2011) and Russo et al. (2014) reported that chromosomes 4D and 7D were linked to grain width in wheat RIL populations. This work mapped the most relevant MTAs for grain length, width, and weight on various chromosomes, namely, 6D, 5D, and 2D. MTAs for several grain characteristics were observed in chromosomal areas 6D 66.4–71.1 cM, 1D 143.5–156.7 cM, and 2D 89.9–92.5 cM (Arora et al., 2017) consistent with the findings in the current study. Due to their direct impact on increasing grain yield, 38 MTAs for grain morphology-related traits and TGW were comparatively more significant. Co-linearity of the MTAs of many traits was observed on chromosomes 1A, 2B, 3A, 3D, and 5B, indicating that these areas were stable (Rasheed et al., 2014).

Grain yield per plant is heavily influenced by grain weight. Simmonds et al. (2014) reported that the effect of a yield MTA on chromosome 6A was driven primarily by increased grain weight, suggesting that enhancing grain weight could contribute to the genetic improvement of wheat yield. Advantageous QTLs for grain weight from common wheat’s diploid D donor have also been identified (Xiang-Zheng et al., 2008). Grain size is a quantitative trait in wheat and is regulated by significant QTLs on most chromosomes of the wheat genome, including 1B, 1D, 2A, 2B, 2D, 3A, 3B, 3D, 4B, 4D, 5A, 5B, 5D, 6A, 6B, 6D, 7A, 7B, and 7D (Cristina et al., 2016). Because of competition for available assimilates, grain size is inversely associated with grain number.

Mir et al. (2012) reported that chromosomes 6A, 1B, 2B, and 6B were linked with grain width in bread wheat genotypes, consistent with the present findings. Grain shape is a quantitative and essential agronomic property with many variables. Many studies have identified MTAs affecting grain shape on multiple chromosomes in popular wheat cultivars. Li et al. (2018) reported an MTA for grain length on chromosome 5B, confirming the present findings. In addition, Wu et al. (2015) observed grain diameter-related MTAs on chromosome 3B, as also reported in the present study. Several MTAs related to grain weight have been identified on chromosome 3D and are available in the literature.

Rasheed et al.(2014) observed the most MTAs (21) on chromosome 2B, followed by 3B (15), and just one MTA on chromosome 6D. The B-genome had the highest number of MTAs (109), followed by the A-genome (60), while the D-genome had the fewest MTAs (28). These results support the current findings.

The results of our study demonstrated the value of genome-wide association mapping for identifying MTAs for grain morphology and yield-related traits in 105 bread wheat genotypes. One pleiotropic locus on chromosome 2D related to Tg-D1 contributed considerably to regulating wheat grain shape (Dvorak et al., 2012). This study discovered the QTL on the short arm of chromosome 2D. MTAs related to grain shape and size are of interest for domestication and breeding programs (Simons et al., 2006; Gegas et al., 2010). Furthermore, the additional stable loci discovered in various contexts are likely novel.

## Conclusion

This study conducted a genome-wide association study (GWAS) through a 90k SNP array of grain morphology and yield-related traits in 105 bread wheat genotypes under control, drought, and heat-stressed conditions. Heritability level was observed from moderate (50.09%) to higher (76.19%). The yield-related traits (TGW and GYP) were significantly correlated with grain morphology-related traits (GW, GC, and GSA) under all examined environmental conditions. This study identified 541 significant MTAs, including 195, 179, and 167 associated with the control, drought, and heat-stressed conditions, respectively. Under control and drought conditions, the pleiotropic loci were *BS00010616_51* and *BS00010868_51* at 186.24 cM and 35.47 cM on chromosomes 7B and 1B, respectively, for the studied traits. The stable SNP (Excalibur_c20796_395) was situated on chromosome 7A at 372.34 cM under the control, drought, and heat-stress conditions. All experimental environments showed multi-trait loci for yield and heat stress tolerance-associated traits on chromosomes 2A, 6A, 7A, 1B, 5B, and 7B. The significant MTAs identified in this study may be useful in marker-assisted selection (MAS) for wheat breeding programs focused on drought and heat tolerance to develop high-yielding wheat genotypes grown under harsh climatic conditions.

## Data Availability

The original contributions presented in the study are included in the Supplementary Material.

## References

[B1] Abdipour M. Ebrahimi M. Izadi-Darbandi A. Mastrangelo A. M. Najafian G. Arshad Y. (2016). Association between grain size and shape and quality traits, and path analysis of thousand grain weight in Iranian bread wheat landraces from different geographic regions. Not. Bot. Horti Agrobot. Cluj. Napoca. 44, 228–236. 10.15835/nbha.44.1.10256

[B2] Ahmed H. G. M.-D. Iqbal M. N. Iqbal M. A. Zeng Y. Ullah A. Iqbal M. (2021). Genome-wide association mapping for stomata and yield indices in bread wheat under water limited conditions. Agronomy 11, 1646. 10.3390/agronomy11081646

[B3] Ahmed H. G. M.-D. Khan A. S. Kashif M. Khan S. (2018). Genetic analysis of yield and physical traits of spring wheat grain. J. Natl. Sci. Found. 46, 23. 10.4038/jnsfsr.v46i1.8262

[B4] Ahmed H. G. M.-D. Sajjad M. Li M. Azmat M. A. Rizwan M. Maqsood R. H. (2019). Selection criteria for drought-tolerant bread wheat genotypes at seedling stage. Sustainability 11, 2584. 10.3390/su11092584

[B5] Ahmed H. G. M.-D. Zeng Y. Yang X. Anwaar H. A. Mansha M. Z. Hanif C. M. S. (2020). Conferring drought-tolerant wheat genotypes through morpho-physiological and chlorophyll indices at seedling stage. Saudi J. Biol. Sci. 27, 2116–2123. 10.1016/j.sjbs.2020.06.019 32714037 PMC7376211

[B6] Ahmed H. Khan A. S. Khan S. H. Kashif M. (2017). Genome wide allelic pattern and genetic diversity of spring wheat genotypes through SSR markers. Int. J. o f Agric. Biol. 19, 1559–1565. 10.17957/IJAB/15.0463

[B8] Arora S. Singh N. Kaur S. Bains N. S. Uauy C. Poland J. (2017). Genome-wide association study of grain architecture in wild wheat Aegilops tauschii. Front. Plant Sci. 8, 886. 10.3389/fpls.2017.00886 28620398 PMC5450224

[B9] Benjamini Y. Hochberg Y. (1995). Controlling the false discovery rate: A practical and powerful approach to multiple testing. J. R. Stat. Soc. Ser. B Methodol. 57, 289–300. 10.1111/j.2517-6161.1995.tb02031.x

[B10] Boz H. Gercekaslan K. E. Karaoğlu M. M. Kotancilar H. G. (2012). Differences in some physical and chemical properties of wheat grains from different parts within the spike. Turkish J. Agric. For. 36, 309–316. 10.3906/tar-1102-41

[B11] Cristina D. Ciuca M. Cornea P. C. (2016). Genetic control of grain size and weight in wheat-where are we now. Sci. Bull. Ser. F. Biotechnol. 20, 27–34.

[B12] Dreisigacker S. Tiwari R. Sheoran S. (2013). Laboratory manual: ICAR-CIMMYT molecular breeding course in wheat. New Delhi: ICAR.

[B13] Dvorak J. Deal K. R. Luo M.-C. You F. M. von Borstel K. Dehghani H. (2012). The origin of spelt and free-threshing hexaploid wheat. J. Hered. 103, 426–441. 10.1093/jhered/esr152 22378960

[B15] Gao L. Yang J. Song S.-j. Xu K. Liu H.-d. Zhang S.-h. (2021). Genome–wide association study of grain morphology in wheat. Euphytica 217, 170–212. 10.1007/s10681-021-02900-1

[B16] Gegas V. C. Nazari A. Griffiths S. Simmonds J. Fish L. Orford S. (2010). A genetic framework for grain size and shape variation in wheat. Plant Cell 22, 1046–1056. 10.1105/tpc.110.074153 20363770 PMC2879751

[B17] Gulnaz S. Zulkiffal M. Sajjad M. Ahmed J. Musa M. Abdullah M. (2019). Identifying Pakistani wheat landraces as genetic resources for yield potential, heat tolerance and rust resistance. Int. J. Agric. Biol. 21, 520–526.

[B18] Guo J. Shi W. Guo J. Yue L. Zhuang L. Zhang W. (2020). Genome-wide association studies on heat stress tolerance during grain development in wheat (*Triticum aestivum* L.) Version 1

[B19] Guo Z. Zhao Y. Röder M. S. Reif J. C. Ganal M. W. Chen D. (2018). Manipulation and prediction of spike morphology traits for the improvement of grain yield in wheat. Sci. Rep. 8, 14435–14510. 10.1038/s41598-018-31977-3 30258057 PMC6158183

[B20] Hansen J. Sato M. Ruedy R. (2012). Perception of climate change. Proc. Natl. Acad. Sci. U. S. A. 109, E2415–E2423. 10.1073/pnas.1205276109 22869707 PMC3443154

[B21] Kuchel H. Williams K. Langridge P. Eagles H. Jefferies S. (2007). Genetic dissection of grain yield in bread wheat. I. QTL analysis. Theor. Appl. Genet. 115, 1029–1041. 10.1007/s00122-007-0629-7 17713755

[B23] Li F. Wen W. He Z. Liu J. Jin H. Cao S. (2018). Genome-wide linkage mapping of yield-related traits in three Chinese bread wheat populations using high-density SNP markers. Theor. Appl. Genet. 131, 1903–1924. 10.1007/s00122-018-3122-6 29858949

[B24] Li Y. Cui Z. Ni Y. Zheng M. Yang D. Jin M. (2016). Plant density effect on grain number and weight of two winter wheat cultivars at different spikelet and grain positions. PloS one 11, e0155351. 10.1371/journal.pone.0155351 27171343 PMC4865215

[B25] Li Y. Zhou R. Wang J. Liao X. Branlard G. Jia J. (2012). Novel and favorable QTL allele clusters for end-use quality revealed by introgression lines derived from synthetic wheat. Mol. Breed. 29, 627–643. 10.1007/s11032-011-9578-6

[B26] Lipka A. E. Tian F. Wang Q. Peiffer J. Li M. Bradbury P. J. (2012). Gapit: Genome association and prediction integrated tool. Bioinformatics 28, 2397–2399. 10.1093/bioinformatics/bts444 22796960

[B27] Mir R. Kumar N. Jaiswal V. Girdharwal N. Prasad M. Balyan H. (2012). Genetic dissection of grain weight in bread wheat through quantitative trait locus interval and association mapping. Mol. Breed. 29, 963–972. 10.1007/s11032-011-9693-4

[B28] Moriondo M. Giannakopoulos C. Bindi M. (2011). Climate change impact assessment: The role of climate extremes in crop yield simulation. Clim. change 104, 679–701. 10.1007/s10584-010-9871-0

[B29] Ni Z. Li H. Zhao Y. Peng H. Hu Z. Xin M. (2018). Genetic improvement of heat tolerance in wheat: Recent progress in understanding the underlying molecular mechanisms. Crop J. 6, 32–41. 10.1016/j.cj.2017.09.005

[B30] Noorka I. R. Teixeira da Silva J. A. (2014). Physical and morphological markers for adaptation of drought-tolerant wheat to arid environments. Pak. J. Agric. Sci. 51, 943.

[B31] Ogunbayo S. Ojo D. Guei R. Oyelakin O. Sanni K. (2005). Phylogenetic diversity and relationships among 40 rice accessions using morphological and RAPDs techniques. Afr. J. Biotechnol. 4, 1234–1244.

[B32] Pinto R. S. Reynolds M. P. Mathews K. L. McIntyre C. L. Olivares-Villegas J.-J. Chapman S. C. (2010). Heat and drought adaptive QTL in a wheat population designed to minimize confounding agronomic effects. Theor. Appl. Genet. 121, 1001–1021. 10.1007/s00122-010-1351-4 20523964 PMC2938441

[B34] Qaseem M. F. Qureshi R. Muqaddasi Q. H. Shaheen H. Kousar R. Röder M. S. (2018). Genome-wide association mapping in bread wheat subjected to independent and combined high temperature and drought stress. PLoS one 13, e0199121. 10.1371/journal.pone.0199121 29949622 PMC6021117

[B35] Rasheed A. Xia X. Ogbonnaya F. Mahmood T. Zhang Z. Mujeeb-Kazi A. (2014). Genome-wide association for grain morphology in synthetic hexaploid wheats using digital imaging analysis. BMC Plant Biol. 14, 128–221. 10.1186/1471-2229-14-128 24884376 PMC4057600

[B36] Ray D. K. Mueller N. D. West P. C. Foley J. A. (2013). Yield trends are insufficient to double global crop production by 2050. PloS one 8, e66428. 10.1371/journal.pone.0066428 23840465 PMC3686737

[B37] Russo M. A. Ficco D. B. M. Laido G. Marone D. Papa R. Blanco A. (2014). A dense durum wheat× T. dicoccum linkage map based on SNP markers for the study of seed morphology. Mol. Breed. 34, 1579–1597. 10.1007/s11032-014-0181-5

[B38] Simmonds J. Scott P. Brinton J. Mestre T. C. Bush M. Del Blanco A. (2016). A splice acceptor site mutation in TaGW2-A1 increases thousand grain weight in tetraploid and hexaploid wheat through wider and longer grains. Theor. Appl. Genet. 129, 1099–1112. 10.1007/s00122-016-2686-2 26883045 PMC4869752

[B39] Simmonds J. Scott P. Leverington-Waite M. Turner A. S. Brinton J. Korzun V. (2014). Identification and independent validation of a stable yield and thousand grain weight QTL on chromosome 6A of hexaploid wheat (*Triticum aestivum* L.) BMC Plant Biol. 14, 191–213. 10.1186/s12870-014-0191-9 25034643 PMC4105860

[B40] Simons K. J. Fellers J. P. Trick H. N. Zhang Z. Tai Y.-S. Gill B. S. (2006). Molecular characterization of the major wheat domestication gene Q. Genetics 172, 547–555. 10.1534/genetics.105.044727 16172507 PMC1456182

[B41] Sorkheh K. Malysheva-Otto L. V. Wirthensohn M. G. Tarkesh-Esfahani S. Martínez-Gómez P. (2008). Linkage disequilibrium, genetic association mapping and gene localization in crop plants. Genet. Mol. Biol. 31, 805–814. 10.1590/s1415-47572008000500001

[B42] Steel R. G. Torrie J. H. (1980). Principles and procedures of statistics: A biometrical approach. New York: McGraw-Hill.

[B43] Su J. Zhang F. Chong X. Song A. Guan Z. Fang W. (2019). Genome-wide association study identifies favorable SNP alleles and candidate genes for waterlogging tolerance in chrysanthemums. Hortic. Res. 6, 21. 10.1038/s41438-018-0101-7 30729011 PMC6355785

[B44] Sukumaran S. Lopes M. Dreisigacker S. Reynolds M. (2018). Genetic analysis of multi-environmental spring wheat trials identifies genomic regions for locus-specific trade-offs for grain weight and grain number. Theor. Appl. Genet. 131, 985–998. 10.1007/s00122-017-3037-7 29218375

[B45] Tadesse W. Ogbonnaya F. Jighly A. Sanchez-Garcia M. Sohail Q. Rajaram S. (2015). Genome-wide association mapping of yield and grain quality traits in winter wheat genotypes. PloS one 10, e0141339. 10.1371/journal.pone.0141339 26496075 PMC4619745

[B46] Team R. (2019). R studio. Boston, MA: Integrated Development for R. RStudio, Inc.

[B47] Wang H. Gao J. Wang H. Zhao C. Li X. Feng D. (2011). Wheat kernel dimensions: How do they contribute to kernel weight at an individual QTL. J. Genet. 90, 409–425. 10.1007/s12041-011-0103-9 22227928

[B48] Wang S. Wong D. Forrest K. Allen A. Chao S. Huang B. E. (2014). Characterization of polyploid wheat genomic diversity using a high‐density 90 000 single nucleotide polymorphism array. Plant Biotechnol. J. 12, 787–796. 10.1111/pbi.12183 24646323 PMC4265271

[B49] Wu Q.-H. Chen Y.-X. Zhou S.-H. Fu L. Chen J.-J. Xiao Y. (2015). High-density genetic linkage map construction and QTL mapping of grain shape and size in the wheat population Yanda1817× Beinong6. PloS one 10, e0118144. 10.1371/journal.pone.0118144 25675376 PMC4326355

[B50] Xiang-Zheng L. Jin W. Rong-Hua Z. Zheng-Long R. Ji-Zeng J. (2008). Mining favorable alleles of QTLs conferring 1000-grain weight from synthetic wheat. A. A. S. 34, 1877–1884. 10.3724/sp.j.1006.2008.01877

[B51] Yan L. Liang F. Xu H. Zhang X. Zhai H. Sun Q. (2017). Identification of QTL for grain size and shape on the D genome of natural and synthetic allohexaploid wheats with near-identical AABB genomes. Front. Plant Sci. 8, 1705. 10.3389/fpls.2017.01705 29075271 PMC5643848

[B52] Zampieri M. Ceglar A. Dentener F. Toreti A. (2017). Wheat yield loss attributable to heat waves, drought and water excess at the global, national and subnational scales. Environ. Res. Lett. 12, 064008. 10.1088/1748-9326/aa723b

