# Supplementary material for: Genome-wide association mapping of bread wheat genotypes using yield and grain morphology-related traits under different environments
Source: Front Genet. 2023 Jan 17;13:1008024. doi: 10.3389/fgene.2022.1008024 (PMC9887163; doi:10.3389/fgene.2022.1008024)
Supplement: Supplementary file 1 [file Table1.docx]

**Table S1: Genotypes code, name and pedigree of 105 spring wheat genotypes**

| Code | Name | Pedigree |
| --- | --- | --- |
| G1 | 9493 | LU26’S’/Pb96 |
| G2 | 9496 | 5039/Rawal87 |
| G3 | 9508 | 5039/Pb96 |
| G4 | 9515 | 4770/Pb76 |
| G5 | 9610 | Pasban90/4943 |
| G6 | 9618 | Pb96/Pasban90 |
| G7 | 9675 | N/A |
| G8 | 9707 | 8060/Rawal87 |
| G9 | 9736 | Inq91/30th SAWSN 30 (1998-99) |
| G10 | 9796 | N/A |
| G11 | 9797 | N/A |
| G12 | 9869 | N/A |
| G13 | 9870 | N/A |
| G14 | 9877 | 9244/PBW222 |
| G15 | 9883 | 9244/Iqbal2000 |
| G16 | 9930 | N/A |
| G17 | 9970 | DN49/ Seher06 |
| G18 | 9889 | 9244/Parwaz94 |
| G19 | 9970 | DN49/Seher06 |
| G20 | 9764 | WLRG 3 1-8 (1993-94)/5039 |
| G21 | BWL-812 | C 591/RN//JN/3/CHR/HD 1941 |
| G22 | PBW-175 | HD2160/4/JN/GAGE//JN/KALYANSONA/3/V-18/C-273; HD-2160/WG-1025; |
| G23 | Anza | LERMA-ROJO-64//NORIN-10/BREVOR/3/3*ANDES-ENANO |
| G24 | PBW 222 | NP 890 /HD 2160 |
| G25 | HD 2307 | HD-2160/116-1-3 |
| G26 | DPW-621-50 | KAUZ//ALTAR-84/(AOS)AWNED-ONAS/3/MILAN/KAUZ/4/HUITES |
| G27 | PBW 343 | Attila |
| G28 | HD 2967 | ALD/COC//URES/HD2160M/HD2278 |
| G29 | BWL-1793 | ND/VG9144 //KAL/BB/3/YCO"S'/4/VEE#5 "S' |
| G30 | BWL-9022 | N/A |
| G31 | BWL-0924 | N/A |
| G32 | C-78711 | N/A |
| G33 | C-252782 | N/A |
| G34 | BWL-1771 | N/A |
| G35 | C-252874 | N/A |
| G36 | C-252803 | N/A |
| G37 | C-118737 | N/A |
| G38 | C-128196 | N/A |
| G39 | 10111 | N/A |
| G40 | C-212185 | N/A |
| G41 | C-32586 | N/A |
| G42 | C-532653 | N/A |
| G43 | C-437081 | N/A |
| G44 | 10115 | N/A |
| G45 | BWL-0814 | N/A |
| G46 | Sonara-64 | N/A |
| G47 | PBN-51 | N/A |
| G48 | C-586642 | N/A |
| G49 | Sakha | INIA-66(S)/NAPO-63 |
| G50 | PBW 621 | KAUZ//ALTAR-84/(AOS)AWNED-ONAS/3/MILAN/KAUZ/4/HUITES |
| G51 | 10117 | N/A |
| G52 | Bareukee | N/A |
| G53 | Redfiled | N/A |
| G54 | Gutha | GAMENYA//GABO*3/KHAPSTEIN(M-146)/3/FALCON*3/CHILE. |
| G55 | Sunstar | CONDOR,AUS/4/2*WW-15/3/STEINWEDEL/YAROSLAV-EMMER//LA-PREVISION |
| G56 | Watan | LU26/HD 2179 |
| G57 | AARI-2011 | SH-88/90A-204//MH97 |
| G58 | Aas-2011 | PRL/PASTOR//2236 |
| G59 | Abadgar-93 | PSN/BOW |
| G60 | Anmol-91 | KVZ/TRM//PTM/ANA |
| G61 | Chakwal-86 | FORLANI/ACC//ANA or Fln/ACS//ANA |
| G62 | Uqab-2000 | CROW'S'/NAC//BOW'S' |
| G63 | Bahawal-97 | PFAU'S'/SERI |
| G64 | Bwp-2000 | AU/UP301//GLL/Sx/3/PEW S/4/MAI S/MAY A S//PEWS |
| G65 | Bakhtawar-94 | Mentana/Mayo//4-11 |
| G66 | Bakhar-2002 | P102/PIMA//F371/TTR/BOW/3/PVN |
| G67 | Bakhtawar-93 | AU/UP301//GLL/SX/3/PEW/4/MAI/MAYA//PEW |
| G68 | Pasban-90 | INIA F66/TH.DISTICHUM//INIAF66/3/GENARO T81 or INIA F66/A.DISTCHUM//INIA66/3/GEN |
| G69 | Bathoor-2008 | URES/JUN//KAUZ |
| G70 | Chakwal-50 | ATTILA/3/HUI/CARC//CHEN/CHTO/4/ATTILA |
| G71 | AS-2002 | KHP/D31708//CMH74A370/3/ENO79/4/R26043/*4NAC |
| G72 | Chakwal-97 | BUC'S'/FCT'S' |
| G73 | Shafaq-2006 | LU 26/HD 2179/ 2*INQALAB 91 |
| G74 | Fakhar-e-Sarhad | NORD-DESPREZ(ND)/VG-9144//KALYANSONA/BLUEBIRD/3/YACO/4/VEERY-5 |
| G75 | Fareed-2006 | PT'S'/3/TOB/LFN//BB/4/BB/HD-832-5//ON/5/G-V/ALD'S'//HPO |
| G76 | FSD-2008 | PBW65/2*Pastor |
| G77 | Millat-2011 | CHENAB2000/INQ-91 |
| G78 | FSD-85 | MAYA/MON//KVZ/TRM |
| G79 | GA 2002 | DWL5023/SNB//SNB |
| G80 | Galaxy-2013 | Pb96/Watan/MH-97 |
| G81 | Gomal-2008 | Attila |
| G82 | Hashim-2008 | JUP/ALD'S'//KLT'S'/3/VEE'S'/6/BEZ//TOB/8156/4/ON/3/6*TH/KF//6*LEE/KF/--------- |
| G83 | Inq-91 | WL 711/CROW "S" |
| G84 | Iqbal-2000 | BURGUS/SORT 12-13//KAL/BB/3/PAK 81 |
| G85 | Kaghan-93 | TTR/JUN |
| G86 | Khyber-87 | KVZ/TRM//PTM/ANA |
| G87 | Kohistan-97 | V-1562//CHRC'S'/HORK/3/KUFRA-I/4/CARP'S'/BJY'S' |
| G88 | Kohinoor-83 | ORE F1 158/FDL//MFN/2*TIBA63/3/COC |
| G89 | Kohsar-95 | PSN/BOW |
| G90 | Lasani-2008 | LUAN/KOH-97 |
| G91 | Ufaq-2002 | V.84133/V83150 |
| G92 | Marvi-2000 | CMH-77A917/PKV 1600//RL6010/6*SKA |
| G93 | Maxi-Pak 65 | PJ/GB55 |
| G94 | Mehran-89 | KVZ/BUHO//KAL/BB |
| G95 | MH-97 | Attila |
| G96 | FSD-83 | FURY//KAL/BB |
| G97 | Mairaj-08 | SPARROW/INIA//V.7394/WL711/3/BAUS |
| G98 | Moomal-2002 | BUC or BUCS/4/TZPP/IRN46 |
| G99 | Margalla-99 | OPATA/BOW'S' |
| G100 | NARC-2009 | INQALAB 91*2/TUKURU |
| G101 | Nifa-barsat 2010 | FRET2 |
| G102 | Nowshera-96 | BUC/FLK//MYNA/VUL |
| G103 | Pak-81 | VEERY. |
| G104 | Parwaz-94 | V.5648/PARULA or V.5648/PRL |
| G105 | BARS-2009 | PFAU/SERI//BOW |

G1-G20 (origin) PBG-UAF= Department of Plant Breeding and Genetics, University of Agriculture, Faisalabad Pakistan.

G21-G55 (origin) Exotic = Foreign (other than Pakistan) spring wheat genotypes.

G56-G105 (origin) Indigenous = Pakistani spring wheat genotypes.

**Table S2: GWAS under Normal Conditions**

| Trait | SNP | Chromosome | Position | P.value | R Square | FDR_Adjusted_P-values |
| --- | --- | --- | --- | --- | --- | --- |
| Grain Length | Kukri_rep_c71356_236 | 7B | 244.31 | 1.99E-14 | 32.71 | 3.84E-10 |
|  | RFL_Contig2949_500 | 5D | 194.19 | 1.99E-14 | 32.39 | 3.84E-10 |
|  | BobWhite_c37236_219 | 3D | 273.05 | 1.75E-13 | 31.53 | 1.69E-09 |
|  | IACX11345 | 1A | 312.46 | 1.75E-13 | 31.24 | 1.69E-09 |
|  | BobWhite_rep_c49910_432 | 7B | 236.25 | 3.12E-12 | 31.03 | 2.41E-08 |
|  | D_contig03323_341 | 7D | 300.22 | 1.92E-09 | 30.76 | 1.10E-05 |
|  | BS00033614_51 | 4B | 168.86 | 1.99E-09 | 30.64 | 1.10E-05 |
|  | Tdurum_contig29645_706 | 4B | 195.17 | 3.64E-09 | 30.63 | 1.75E-05 |
|  | Tdurum_contig92931_882 | 4B | 168.86 | 8.89E-09 | 30.63 | 3.81E-05 |
|  | BS00081631_51 | 4B | 144.06 | 1.14E-08 | 30.63 | 3.97E-05 |
|  | Tdurum_contig25413_208 | 7B | 244.31 | 1.14E-08 | 30.63 | 3.97E-05 |
|  | RFL_Contig467_431 | 7B | 244.31 | 1.30E-08 | 30.63 | 3.97E-05 |
|  | BS00021986_51 | 4B | 163.94 | 1.53E-08 | 30.63 | 3.97E-05 |
|  | BobWhite_c44567_96 | 2D | 160.4 | 1.92E-08 | 30.63 | 3.97E-05 |
|  | BS00009439_51 | 4B | 176.21 | 2.28E-08 | 30.63 | 3.97E-05 |
|  | BS00097391_51 | 4B | 176.21 | 2.28E-08 | 30.63 | 3.97E-05 |
|  | Tdurum_contig12116_518 | 4B | 176.21 | 2.28E-08 | 30.39 | 3.97E-05 |
|  | BS00011859_51 | 4B | 176.21 | 2.28E-08 | 30.39 | 3.97E-05 |
|  | TA002011-0754 | 4B | 176.21 | 2.28E-08 | 30.39 | 3.97E-05 |
|  | Tdurum_contig58145_743 | 4B | 176.21 | 2.28E-08 | 28.73 | 3.97E-05 |
|  | GENE-0173_168 | 1B | 206.01 | 2.47E-08 | 27.98 | 3.97E-05 |
|  | GENE-0173_309 | 1B | 206.01 | 2.47E-08 | 27.76 | 3.97E-05 |
|  | Tdurum_contig60547_330 | 1B | 206.01 | 2.47E-08 | 27.63 | 3.97E-05 |
|  | Tdurum_contig60547_129 | 1B | 208.49 | 2.47E-08 | 26.62 | 3.97E-05 |
|  | Excalibur_c2056_130 | 4B | 176.21 | 4.17E-08 | 26.32 | 4.47E-05 |
|  | Excalibur_c28382_336 | 4B | 176.21 | 4.17E-08 | 25.82 | 4.47E-05 |
|  | Kukri_c26943_862 | 4B | 176.21 | 4.17E-08 | 25.26 | 4.47E-05 |
|  | Tdurum_contig76677_265 | 4B | 176.21 | 4.17E-08 | 24.71 | 4.47E-05 |
|  | BS00011851_51 | 4B | 176.21 | 4.17E-08 | 24.19 | 4.47E-05 |
|  | BS00084904_51 | 4B | 176.21 | 4.17E-08 | 23.84 | 4.47E-05 |
|  | Kukri_c2913_231 | 4B | 176.21 | 4.17E-08 | 23.61 | 4.47E-05 |
|  | Kukri_c42458_499 | 4B | 176.21 | 4.17E-08 | 23.47 | 4.47E-05 |
|  | TA001928-1401 | 4B | 176.21 | 4.17E-08 | 23.06 | 4.47E-05 |
|  | Tdurum_contig10187_1271 | 4B | 176.21 | 4.17E-08 | 22.87 | 4.47E-05 |
|  | Tdurum_contig50123_97 | 4B | 176.21 | 4.17E-08 | 22.75 | 4.47E-05 |
|  | wsnp_BE422566B_Ta_1_2 | 4B | 176.21 | 4.17E-08 | 22.75 | 4.47E-05 |
|  | BS00044443_51 | 7B | 236.25 | 4.44E-08 | 22.71 | 4.63E-05 |
|  | Tdurum_contig47605_1362 | 7B | 245.61 | 6.04E-08 | 22.69 | 6.13E-05 |
|  | CAP11_c601_120 | 4B | 168.86 | 6.21E-08 | 22.27 | 6.14E-05 |
|  | TA002925-3757 | 4B | 176.21 | 6.38E-08 | 22.07 | 6.15E-05 |
|  | Excalibur_c1066_303 | 1A | 315.94 | 7.27E-08 | 21.88 | 6.83E-05 |
|  | BobWhite_c27251_77 | 7A | 458.76 | 8.34E-08 | 21.76 | 7.66E-05 |
|  | Tdurum_contig82378_561 | 4B | 163.94 | 1.06E-07 | 21.76 | 9.06E-05 |
|  | BS00022047_51 | 4B | 168.86 | 1.06E-07 | 21.44 | 9.06E-05 |
|  | BS00022785_51 | 4B | 163.94 | 1.06E-07 | 21.32 | 9.06E-05 |
|  | BS00011338_51 | 4B | 163.94 | 1.32E-07 | 21.19 | 0.000109563 |
|  | Tdurum_contig48847_852 | 7B | 234.95 | 1.39E-07 | 20.93 | 0.000109563 |
|  | RAC875_rep_c107704_155 | 7B | 234.95 | 1.39E-07 | 20.90 | 0.000109563 |
|  | Tdurum_contig48847_669 | 7B | 234.95 | 1.39E-07 | 20.78 | 0.000109563 |
|  | Tdurum_contig76677_1142 | 4B | 176.21 | 1.53E-07 | 20.78 | 0.000117929 |
| Grain Width | BobWhite_c35520_397 | 1B | 230.29 | 7.93E-07 | 30.77 | 0.012265047 |
|  | wsnp_Ex_c16008_24427927 | 6B | 73.95 | 8.90E-07 | 30.47 | 0.012265047 |
|  | Ex_c13742_757 | 1B | 206.01 | 9.55E-07 | 30.29 | 0.012265047 |
|  | BS00065696_51 | 4B | 17.47 | 3.06E-06 | 27.32 | 0.024165848 |
|  | IAAV3082 | 1B | 230.29 | 3.13E-06 | 27.26 | 0.024165848 |
|  | wsnp_Ex_rep_c66643_64952627 | 1B | 224.75 | 4.86E-06 | 26.16 | 0.031201339 |
|  | wsnp_Ex_c12774_20272038 | 1B | 230.29 | 5.93E-06 | 25.66 | 0.031635146 |
|  | RAC875_c92464_53 | 1B | 224.75 | 6.57E-06 | 25.41 | 0.031635146 |
|  | RAC875_rep_c70937_641 | 1B | 230.29 | 7.49E-06 | 25.09 | 0.032073906 |
|  | D_contig30716_172 | 7D | 219.04 | 1.22E-05 | 23.90 | 0.044958956 |
|  | Excalibur_s112663_236 | 1A | 481.08 | 1.60E-05 | 23.23 | 0.044958956 |
|  | IAAV5455 | 1B | 240.49 | 1.75E-05 | 23.03 | 0.044958956 |
|  | RAC875_rep_c70937_1065 | 1B | 230.29 | 1.75E-05 | 23.03 | 0.044958956 |
|  | wsnp_Ex_rep_c67942_66669791 | 1B | 230.29 | 1.75E-05 | 23.03 | 0.044958956 |
|  | BS00083072_51 | 1B | 224.75 | 2.00E-05 | 22.70 | 0.044958956 |
|  | Excalibur_c7035_155 | 1B | 224.75 | 2.03E-05 | 22.67 | 0.044958956 |
|  | Excalibur_c57881_200 | 1B | 230.29 | 2.25E-05 | 22.42 | 0.044958956 |
|  | BS00097477_51 | 1B | 215.54 | 2.29E-05 | 22.37 | 0.044958956 |
|  | Kukri_c41217_154 | 2A | 348.36 | 2.55E-05 | 22.12 | 0.044958956 |
|  | Ra_c43931_376 | 2A | 348.36 | 2.55E-05 | 22.12 | 0.044958956 |
|  | RFL_Contig3916_275 | 2A | 341.14 | 2.55E-05 | 22.12 | 0.044958956 |
|  | Ex_c19669_309 | 7B | 508.97 | 2.57E-05 | 22.10 | 0.044958956 |
|  | Ex_c67541_975 | 1B | 230.29 | 2.94E-05 | 21.78 | 0.047288448 |
|  | wsnp_Ex_c2278_4270123 | 1B | 226.95 | 2.94E-05 | 21.78 | 0.047288448 |
|  | RAC875_rep_c111001_187 | 1B | 244.91 | 3.11E-05 | 21.64 | 0.048006979 |
| grain diameter | Tdurum_contig48049_705 | 4A | 160.42 | 4.96E-06 | 28.03 | 0.000137515 |
|  | BS00013824_51 | 1B | 208.49 | 2.26E-05 | 24.24 | 0.000143082 |
|  | Tdurum_contig46313_394 | 4B | 211.4 | 4.96E-05 | 22.33 | 0.000143082 |
|  | Tdurum_contig59440_1621 | 7B | 236.25 | 5.78E-05 | 21.96 | 0.00015267 |
|  | Ra_c327_1572 | 3B | 100.36 | 8.14E-05 | 21.15 | 0.000155231 |
|  | JG_c2645_107 | 3A | 391.81 | 0.00016 | 19.53 | 0.000155231 |
|  | Excalibur_c55800_202 | 6B | 148.84 | 0.00021 | 18.96 | 0.000193052 |
|  | Tdurum_contig83933_258 | 6A | 190.27 | 0.00022 | 18.86 | 0.00021067 |
|  | Excalibur_c65109_60 | 5B | 175.82 | 0.00028 | 18.30 | 0.000259117 |
|  | wsnp_Ku_rep_c72211_71920520 | 5B | 175.82 | 0.00028 | 18.30 | 0.000305673 |
|  | Kukri_c14877_303 | 6A | 190.27 | 0.00031 | 18.04 | 0.000336191 |
|  | Tdurum_contig13784_824 | 5B | 168.1 | 0.00033 | 17.92 | 0.000336191 |
|  | Excalibur_c41898_218 | 5B | 168.1 | 0.00033 | 17.92 | 0.000481906 |
|  | GENE-4403_405 | 7B | 244.31 | 0.00033 | 17.86 | 0.000577379 |
|  | wsnp_Ex_rep_c69631_68583363 | 5B | 175.82 | 0.00039 | 17.48 | 0.000645147 |
|  | CAP7_c6531_308 | 1A | 241.88 | 0.00041 | 17.41 | 0.000895949 |
|  | Kukri_c36317_305 | 5B | 175.82 | 0.00041 | 17.40 | 0.000988729 |
|  | Excalibur_c766_771 | 6B | 148.84 | 0.00045 | 17.19 | 0.001061661 |
|  | wsnp_CAP11_c905_550912 | 2A | 341.14 | 0.00045 | 17.17 | 0.001069176 |
|  | BS00010576_51 | 6A | 190.27 | 0.00047 | 17.10 | 0.001069176 |
|  | Tdurum_contig52096_270 | 7B | 497.57 | 0.00048 | 17.04 | 0.001069176 |
|  | Kukri_rep_c101182_788 | 5B | 143.28 | 0.0005 | 16.94 | 0.001069176 |
|  | Tdurum_contig10205_807 | 6A | 190.27 | 0.00054 | 16.78 | 0.001069176 |
|  | Tdurum_contig25337_77 | 1B | 212.19 | 0.00055 | 16.72 | 0.001069176 |
|  | BS00010868_51 | 1B | 35.47 | 0.00062 | 16.48 | 0.001069176 |
|  | D_contig65543_191 | 5D | 189.99 | 0.00066 | 16.34 | 0.001069176 |
|  | Tdurum_contig52096_330 | 7B | 501.16 | 0.0007 | 16.18 | 0.001069176 |
|  | Excalibur_c20796_395 | 7A | 372.34 | 0.00082 | 15.85 | 0.001069176 |
|  | wsnp_RFL_Contig4424_5193532 | 6A | 198.23 | 0.00083 | 15.81 | 0.001069176 |
|  | Tdurum_contig44261_127 | 7B | 195.74 | 0.00087 | 15.71 | 0.001136333 |
|  | BobWhite_c10483_531 | 6A | 198.23 | 0.00091 | 15.62 | 0.001136333 |
|  | Ku_c105902_308 | 3B | 275.44 | 0.00093 | 15.56 | 0.001136333 |
|  | wsnp_Ex_c2727_5053747 | 5B | 212.2 | 0.00097 | 15.47 | 0.00220335 |
|  | wsnp_RFL_Contig1570_778491 | 5B | 212.38 | 0.00097 | 15.47 | 0.002953831 |
|  | RAC875_c30829_1711 | 5B | 179.66 | 0.00097 | 15.47 | 0.003196545 |
|  | wsnp_Ex_c1314_2513802 | 5B | 212.38 | 0.00097 | 15.47 | 0.003342721 |
|  | wsnp_RFL_Contig2504_2093982 | 5B | 179.66 | 0.00097 | 15.47 | 0.005028639 |
|  | Tdurum_contig93156_239 | 4A | 497.16 | 0.00099 | 15.42 | 0.005621909 |
| Grain roundness | Tdurum_contig100702_265 | 4A | 542.67 | 1.94E-05 | 21.32 | 0.006874582 |
|  | Excalibur_c48047_90 | 3A | 321.73 | 6.53E-05 | 18.39 | 0.008620596 |
|  | BobWhite_c19429_95 | 7B | 427.7 | 0.0003 | 14.81 | 0.010760628 |
|  | RFL_Contig1445_1192 | 2B | 343.22 | 0.00033 | 14.61 | 0.013310231 |
|  | Kukri_c46740_226 | 3D | 0 | 0.00037 | 14.38 | 0.015245581 |
|  | Ex_c10068_1509 | 2A | 479.11 | 0.00054 | 13.51 | 0.016717607 |
|  | D_GDEEGVY01CQJ66_272 | 7A | 375.14 | 0.00093 | 12.30 | 0.017510726 |
|  | Kukri_c13134_132 | 1D | 5.63 | 0.00094 | 12.28 | 0.020703921 |
| grain circumference | Tdurum_contig82633_313 | 4B | 176.21 | 3.07E-07 | 32.58 | 0.011821692 |
|  | BS00000487_51 | 1B | 64.23 | 3.17E-06 | 26.49 | 0.024387915 |
|  | Kukri_c15594_386 | 7A | 360.88 | 4.52E-06 | 25.59 | 0.024387915 |
|  | Tdurum_contig8741_194 | 6D | 352.32 | 4.52E-06 | 25.59 | 0.024387915 |
|  | GENE-1343_482 | 2A | 157.96 | 4.78E-06 | 25.45 | 0.024387915 |
|  | CAP7_c4676_94 | 2A | 24.69 | 4.98E-06 | 25.34 | 0.024387915 |
|  | BS00045171_51 | 2A | 162.06 | 4.98E-06 | 25.34 | 0.024387915 |
|  | Kukri_c40121_373 | 2A | 388.1 | 5.39E-06 | 25.15 | 0.024387915 |
|  | RAC875_c33757_237 | 3A | 347.91 | 5.69E-06 | 25.01 | 0.024387915 |
|  | JG_c7136_416 | 7D | 301.64 | 1.24E-05 | 23.09 | 0.047656263 |
|  | GENE-4937_537 | 2D | 298.38 | 1.75E-05 | 22.24 | 0.057022288 |
|  | Tdurum_contig42087_1199 | 6B | 229.64 | 1.85E-05 | 22.10 | 0.057022288 |
|  | BS00068396_51 | 2A | 157.96 | 1.92E-05 | 22.01 | 0.057022288 |
|  | RAC875_c103017_302 | 4B | 263.93 | 2.20E-05 | 21.68 | 0.057544168 |
|  | BS00067342_51 | 2A | 86.88 | 2.24E-05 | 21.64 | 0.057544168 |
| grain surface area | BS00035630_51 | 7B | 172.1 | 4.25E-05 | 19.42 | 0.022241228 |
|  | IACX1805 | 7B | 261.77 | 5.63E-05 | 18.74 | 0.023033053 |
|  | Tdurum_contig43966_1107 | 7B | 261.77 | 5.63E-05 | 18.74 | 0.023033053 |
|  | Tdurum_contig43966_813 | 7B | 261.77 | 5.63E-05 | 18.74 | 0.023120104 |
|  | tplb0039c07_334 | 7B | 258.64 | 5.63E-05 | 18.74 | 0.023154522 |
|  | Kukri_c38676_251 | 7B | 261.77 | 5.63E-05 | 18.74 | 0.027515389 |
|  | Kukri_c11154_1723 | 5B | 126.02 | 0.00011 | 17.21 | 0.029747157 |
|  | RAC875_c38274_239 | 5A | 78.41 | 0.00015 | 16.44 | 0.03206866 |
|  | BobWhite_c15796_315 | 7B | 229.43 | 0.00028 | 14.96 | 0.033200167 |
|  | GENE-4720_644 | 7B | 228.36 | 0.00028 | 14.96 | 0.033200167 |
|  | GENE-4790_279 | 7B | 228.36 | 0.00028 | 14.96 | 0.037758423 |
|  | RFL_Contig2540_306 | 7B | 228.36 | 0.00028 | 14.96 | 0.039586513 |
|  | Tdurum_contig42813_285 | 7B | 229.43 | 0.00028 | 14.96 | 0.041466668 |
|  | Tdurum_contig93266_145 | 7B | 229.43 | 0.00028 | 14.96 | 0.046226388 |
|  | BobWhite_c16787_205 | 7B | 229.43 | 0.00028 | 14.96 | 0.046390497 |
|  | BobWhite_c7907_657 | 7B | 228.36 | 0.00028 | 14.96 | 0.047673229 |
|  | BS00028793_51 | 7B | 228.36 | 0.00028 | 14.96 | 0.047673229 |
|  | Excalibur_c22340_449 | 7A | 398.79 | 0.00028 | 14.96 | 0.05958831 |
| thousand grain weight | CAP11_c2285_104 | 1A | 357.33 | 2.71E-06 | 26.26 | 0.035228317 |
|  | BS00065853_51 | 3A | 271.98 | 3.20E-06 | 25.83 | 0.035228317 |
|  | RAC875_rep_c91551_80 | 4B | 182.55 | 1.48E-05 | 21.98 | 0.035228317 |
|  | Excalibur_rep_c101791_575 | 4B | 182.46 | 3.38E-05 | 19.97 | 0.035228317 |
|  | RAC875_c5351_780 | 4B | 182.55 | 3.38E-05 | 19.97 | 0.035228317 |
|  | RAC875_rep_c96091_211 | 2B | 309.48 | 3.38E-05 | 19.97 | 0.035228317 |
|  | TA006298-0500 | 4B | 182.55 | 3.38E-05 | 19.97 | 0.035228317 |
|  | CAP11_c991_160 | 6B | 245.09 | 3.38E-05 | 19.97 | 0.035228317 |
|  | D_GDS7LZN02GGGLS_113 | 4D | 175.3 | 3.38E-05 | 19.97 | 0.035228317 |
|  | Excalibur_c27716_544 | 2B | 277.23 | 3.38E-05 | 19.97 | 0.035228317 |
|  | GENE-2957_288 | 4B | 182.55 | 3.38E-05 | 19.97 | 0.035228317 |
|  | Kukri_c27822_55 | 4B | 182.55 | 3.38E-05 | 19.97 | 0.035228317 |
|  | Kukri_c65511_1057 | 1A | 202.05 | 3.38E-05 | 19.97 | 0.035228317 |
|  | Kukri_rep_c110025_90 | 4B | 182.55 | 3.38E-05 | 19.97 | 0.035228317 |
|  | RFL_Contig4542_1281 | 2B | 277.23 | 3.38E-05 | 19.97 | 0.035228317 |
|  | Tdurum_contig10300_400 | 4B | 183.6 | 3.38E-05 | 19.97 | 0.035228317 |
|  | Tdurum_contig13044_268 | 1B | 238.2 | 3.38E-05 | 19.97 | 0.035228317 |
|  | Tdurum_contig42038_2184 | 4B | 182.55 | 3.38E-05 | 19.97 | 0.035228317 |
|  | Tdurum_contig48396_341 | 1B | 208.49 | 3.38E-05 | 19.97 | 0.035228317 |
|  | Tdurum_contig52356_517 | 4B | 182.55 | 3.38E-05 | 19.97 | 0.035228317 |
|  | wsnp_Ku_c50833_56310208 | 3A | 321.73 | 3.38E-05 | 19.97 | 0.035228317 |
|  | Tdurum_contig50731_961 | 5B | 400.89 | 4.07E-05 | 19.52 | 0.041271175 |
| Grain Yield per plant | Tdurum_contig48231_1233 | 4A | 191.56 | 1.95E-07 | 34.04 | 0.003760472 |
|  | Excalibur_c20796_395 | 7A | 372.34 | 3.44E-07 | 32.51 | 0.004426703 |
|  | Tdurum_contig46313_394 | 4B | 211.4 | 3.31E-06 | 26.64 | 0.027025257 |
|  | Ex_c8261_2309 | 3D | 416.72 | 3.51E-06 | 26.50 | 0.027025257 |
|  | GENE-4403_405 | 7B | 244.31 | 1.27E-05 | 23.31 | 0.035228317 |
|  | Tdurum_contig13784_824 | 5B | 168.1 | 1.50E-05 | 22.89 | 0.035228317 |
|  | Excalibur_c41898_218 | 5B | 168.1 | 1.50E-05 | 22.89 | 0.035228317 |
|  | Excalibur_c766_771 | 6B | 148.84 | 2.80E-05 | 21.38 | 0.035228317 |
|  | Ku_c16468_146 | 2B | 368.5 | 2.95E-05 | 21.26 | 0.035228317 |
|  | Tdurum_contig6949_697 | 1B | 208.49 | 3.46E-05 | 20.88 | 0.035228317 |
|  | Tdurum_contig44261_127 | 7B | 195.74 | 4.17E-05 | 20.43 | 0.035228317 |
|  | BS00010616_51 | 7B | 186.24 | 5.43E-05 | 19.81 | 0.035228317 |
|  | RAC875_c30829_1711 | 5B | 179.66 | 5.59E-05 | 19.74 | 0.035228317 |
|  | wsnp_Ex_c1314_2513802 | 5B | 212.38 | 5.59E-05 | 19.74 | 0.035228317 |
|  | wsnp_RFL_Contig2504_2093982 | 5B | 179.66 | 5.59E-05 | 19.74 | 0.035228317 |
|  | wsnp_Ex_c2727_5053747 | 5B | 212.2 | 5.59E-05 | 19.74 | 0.035228317 |
|  | wsnp_RFL_Contig1570_778491 | 5B | 212.38 | 5.59E-05 | 19.74 | 0.035228317 |
|  | wsnp_BE444579B_Ta_2_1 | 3B | 558.39 | 9.36E-05 | 18.54 | 0.035228317 |
|  | BobWhite_c34267_459 | 1B | 273.29 | 9.87E-05 | 18.41 | 0.041271175 |

**Table S3: GWAS under Drought stressed Conditions**

| Trait | SNP | Chromosome | Position | P.value | R% | FDR_Adjusted_P-values |
| --- | --- | --- | --- | --- | --- | --- |
| Grain length | Kukri_c45439_457 | 5A | 78.15 | 7.60E-06 | 26.62 | 0.000143082 |
|  | GENE-0025_120 | 1B | 201.25 | 6.36E-05 | 21.64 | 0.000143082 |
|  | RAC875_s117925_244 | 5A | 77.77 | 9.37E-05 | 20.75 | 0.00015267 |
|  | Excalibur_c114072_548 | 5B | 412.14 | 0.0001012 | 20.58 | 0.000155231 |
|  | Tdurum_contig48049_705 | 4A | 160.42 | 0.000102 | 20.56 | 0.000155231 |
|  | tplb0026b15_350 | 1B | 195.12 | 0.0002238 | 18.81 | 0.000193052 |
|  | Ex_c8261_2309 | 3D | 416.72 | 0.0002423 | 18.63 | 0.00021067 |
|  | Excalibur_rep_c96693_837 | 1B | 201.25 | 0.0002501 | 18.56 | 0.000259117 |
|  | RAC875_c1914_942 | 1B | 205.25 | 0.0003158 | 18.05 | 0.000305673 |
|  | Tdurum_contig1015_131 | 3D | 292.51 | 0.0003651 | 17.73 | 0.000336191 |
|  | Tdurum_contig100344_184 | 1B | 201.25 | 0.0004001 | 17.54 | 0.000336191 |
|  | Ra_c5508_706 | 4B | 236.17 | 0.0005022 | 17.04 | 0.000481906 |
|  | Tdurum_contig100344_160 | 1B | 201.25 | 0.0005045 | 17.03 | 0.000577379 |
|  | BS00090003_51 | 1B | 201.25 | 0.0005155 | 16.99 | 0.000645147 |
|  | wsnp_Ku_c9144_15396815 | 1B | 205.45 | 0.0005212 | 16.96 | 0.000895949 |
|  | Kukri_c11707_160 | 1B | 195.12 | 0.0005405 | 16.88 | 0.000988729 |
|  | GENE-2162_650 | 1B | 201.25 | 0.0005472 | 16.86 | 0.001061661 |
|  | RAC875_c64610_285 | 7D | 358.28 | 0.0006074 | 16.63 | 0.001069176 |
|  | Ku_c8810_903 | 1B | 142.58 | 0.0006263 | 16.57 | 0.001069176 |
|  | D_contig33799_445 | 1B | 195.12 | 0.0006352 | 16.54 | 0.001069176 |
|  | IAAV7602 | 1B | 195.12 | 0.0006843 | 16.38 | 0.001069176 |
|  | Kukri_rep_c108883_577 | 1B | 142.58 | 0.0006883 | 16.37 | 0.001069176 |
|  | wsnp_Ex_c26285_35531324 | 4B | 238.43 | 0.0007166 | 16.28 | 0.001069176 |
|  | CAP12_c715_102 | 1B | 195.12 | 0.0007268 | 16.25 | 0.001069176 |
|  | RAC875_rep_c69176_194 | 1B | 201.25 | 0.0007889 | 16.08 | 0.001069176 |
|  | Tdurum_contig87227_108 | 1B | 201.25 | 0.0007889 | 16.08 | 0.001069176 |
| Grain Width | BobWhite_c43880_73 | 4D | 159.35 | 3.17E-07 | 31.92 | 0.010760628 |
|  | CAP8_rep_c5023_658 | 4D | 119.73 | 2.41E-05 | 20.79 | 0.013310231 |
|  | wsnp_CAP11_c356_280910 | 4D | 119.73 | 2.41E-05 | 20.79 | 0.015245581 |
|  | Excalibur_c15258_163 | 5A | 99.71 | 3.17E-05 | 20.13 | 0.016717607 |
|  | Excalibur_c7338_242 | 7B | 427.7 | 0.0002225 | 15.53 | 0.017510726 |
|  | Kukri_c11959_587 | 7B | 427.7 | 0.0002225 | 15.53 | 0.020703921 |
|  | D_contig28294_88 | 4B | 167.74 | 0.0003396 | 14.56 | 0.022241228 |
|  | Kukri_c32139_2473 | 3D | 416.72 | 0.0003596 | 14.43 | 0.023033053 |
|  | Kukri_c16814_103 | 7B | 427.7 | 0.0003931 | 14.23 | 0.023033053 |
|  | IAAV5117 | 4B | 208.51 | 0.0006762 | 13.01 | 0.023120104 |
|  | Kukri_c865_59 | 5A | 463.65 | 0.0009049 | 12.37 | 0.023154522 |
|  | Excalibur_c7338_563 | 7B | 427.7 | 0.0009906 | 12.17 | 0.027515389 |
| Grain Diameter | Tdurum_contig48049_705 | 4A | 160.42 | 1.89E-05 | 21.48 | 0.023033053 |
|  | BobWhite_c5441_151 | 1B | 105.85 | 2.89E-05 | 20.44 | 0.023120104 |
|  | Excalibur_c30462_100 | 5A | 36 | 3.27E-05 | 20.14 | 0.023154522 |
|  | Excalibur_c20796_395 | 7A | 372.34 | 3.96E-05 | 19.68 | 0.027515389 |
|  | BobWhite_c11000_782 | 3D | 416.72 | 4.49E-05 | 19.38 | 0.029747157 |
|  | D_GBUVHFX01EH6C6_52 | 5A | 77.77 | 5.60E-05 | 18.85 | 0.03206866 |
|  | Ra_c16172_309 | 6D | 330.34 | 7.62E-05 | 18.12 | 0.033200167 |
|  | IACX11026 | 6D | 330.34 | 7.62E-05 | 18.12 | 0.033200167 |
|  | Kukri_c45439_457 | 5A | 78.15 | 0.000145 | 16.61 | 0.037758423 |
|  | BS00022206_51 | 6D | 335.87 | 0.0004199 | 14.18 | 0.039586513 |
|  | BobWhite_c15644_236 | 5A | 78.15 | 0.0004204 | 14.18 | 0.041466668 |
|  | D_F1BEJMU02FXGZS_209 | 2D | 120.51 | 0.0004702 | 13.92 | 0.046226388 |
|  | Excalibur_c3526_106 | 6D | 217.41 | 0.0005071 | 13.75 | 0.046390497 |
|  | BS00009369_51 | 5A | 421.21 | 0.0005086 | 13.75 | 0.047673229 |
|  | BobWhite_c17445_83 | 5A | 484.57 | 0.0007178 | 12.98 | 0.047673229 |
|  | Ra_c69621_237 | 5B | 320.72 | 0.0007782 | 12.80 | 0.047673229 |
|  | CAP12_c4007_435 | 1B | 246.08 | 0.0008413 | 12.63 | 0.05106091 |
|  | D_contig37543_277 | 5A | 64.47 | 0.0008623 | 12.57 | 0.052512344 |
|  | BS00080570_51 | 2A | 31.48 | 0.0008726 | 12.55 | 0.058852315 |
|  | Kukri_c80999_191 | 5A | 77.82 | 0.0008983 | 12.48 | 0.05958831 |
|  | BS00067501_51 | 5B | 222.57 | 0.0009909 | 12.27 | 0.05958831 |
| Grain Roundness | D_contig57523_172 | 2D | 183.76 | 5.73E-06 | 25.27 | 0.000259117 |
|  | Excalibur_c33002_123 | 7D | 375.36 | 3.20E-05 | 21.07 | 0.000305673 |
|  | Excalibur_c25991_184 | 7B | 169.41 | 0.0001143 | 18.08 | 0.000336191 |
|  | Tdurum_contig59449_1462 | 1B | 240.49 | 0.0001194 | 17.98 | 0.000336191 |
|  | IACX419 | 6B | 237.57 | 0.000121 | 17.95 | 0.000481906 |
|  | Kukri_c27958_334 | 6A | 153.27 | 0.0001337 | 17.72 | 0.000577379 |
|  | RAC875_c26328_75 | 7B | 169.41 | 0.000227 | 16.51 | 0.000645147 |
|  | BS00093857_51 | 3D | 240.59 | 0.0003122 | 15.79 | 0.000895949 |
|  | BS00095640_51 | 3D | 240.59 | 0.0003122 | 15.79 | 0.000988729 |
|  | BS00096882_51 | 2A | 385.17 | 0.0003122 | 15.79 | 0.001061661 |
|  | D_contig24171_152 | 6D | 191.27 | 0.0003122 | 15.79 | 0.001069176 |
|  | D_F5MV3MU01DWT7N_151 | 3D | 432.46 | 0.0003122 | 15.79 | 0.001069176 |
|  | D_GCE8AKX01CWZ8Z_144 | 6D | 191.27 | 0.0003122 | 15.79 | 0.001069176 |
|  | Ex_c54098_174 | 5A | 78.01 | 0.0003122 | 15.79 | 0.001069176 |
|  | Excalibur_c22503_628 | 3D | 432.46 | 0.0003122 | 15.79 | 0.001069176 |
|  | Excalibur_c33112_226 | 7A | 683.43 | 0.0003122 | 15.79 | 0.001069176 |
|  | Excalibur_rep_c69159_392 | 5D | 177.52 | 0.0003122 | 15.79 | 0.001069176 |
|  | Ku_c18550_1388 | 3D | 432.46 | 0.0003122 | 15.79 | 0.001069176 |
|  | Ku_c6501_507 | 5D | 177.52 | 0.0003122 | 15.79 | 0.001069176 |
|  | RAC875_c30984_307 | 2A | 385.17 | 0.0003122 | 15.79 | 0.001069176 |
|  | RAC875_rep_c72040_1809 | 7D | 309.36 | 0.0003122 | 15.79 | 0.001069176 |
|  | wsnp_Ex_rep_c66524_64798744 | 2B | 319.82 | 0.0003122 | 15.79 | 0.001136333 |
|  | wsnp_Ex_rep_c72527_70882805 | 2B | 210.62 | 0.0003122 | 15.79 | 0.001136333 |
|  | D_contig22507_191 | 6D | 191.27 | 0.0003122 | 15.79 | 0.001136333 |
|  | D_GCE8AKX01CTXDI_46 | 6D | 191.27 | 0.0003122 | 15.79 | 0.00220335 |
|  | Ex_c6013_558 | 5D | 185.6 | 0.0003122 | 15.79 | 0.002953831 |
|  | BS00050993_51 | 7B | 169.41 | 0.0004174 | 15.14 | 0.003196545 |
|  | Tdurum_contig45927_968 | 5A | 626.85 | 0.0004314 | 15.06 | 0.003342721 |
|  | wsnp_Ex_rep_c93362_82371891 | 2A | 177.91 | 0.0004567 | 14.94 | 0.005028639 |
|  | IAAV5655 | 5D | 177.52 | 0.0005925 | 14.36 | 0.005621909 |
|  | Kukri_c20060_350 | 6D | 249.83 | 0.0005925 | 14.36 | 0.006874582 |
|  | Ra_c74015_289 | 5D | 177.52 | 0.0005925 | 14.36 | 0.008620596 |
|  | wsnp_Ku_c11846_19263340 | 6B | 291.03 | 0.0005925 | 14.36 | 0.010760628 |
|  | RAC875_c62936_139 | 2A | 176.43 | 0.0008475 | 13.57 | 0.013310231 |
|  | wsnp_RFL_Contig366_3864231 | 2A | 217.13 | 0.0008525 | 13.56 | 0.015245581 |
|  | BS00087197_51 | 7B | 508.33 | 0.000904 | 13.43 | 0.016717607 |
|  | Kukri_c31508_91 | 2A | 176.43 | 0.0009729 | 13.27 | 0.017510726 |
| Grain Circumferences | Excalibur_c39284_949 | 1B | 510.03 | 4.58E-06 | 24.97 | 0.023033053 |
|  | Excalibur_c5888_169 | 1B | 509.37 | 3.15E-05 | 20.20 | 0.023033053 |
|  | BS00072620_51 | 2B | 124 | 4.60E-05 | 19.29 | 0.023120104 |
|  | Kukri_c59535_186 | 1B | 509.37 | 8.15E-05 | 17.93 | 0.023154522 |
|  | BS00022053_51 | 7B | 306.41 | 0.000319 | 14.76 | 0.027515389 |
|  | wsnp_Ku_c7297_12596001 | 2B | 368.5 | 0.0004943 | 13.77 | 0.029747157 |
|  | Kukri_c14891_64 | 7B | 303.32 | 0.0005302 | 13.62 | 0.03206866 |
|  | BS00066873_51 | 7B | 303.32 | 0.0005505 | 13.53 | 0.033200167 |
|  | wsnp_RFL_Contig2996_2877869 | 5D | 277.56 | 0.0005544 | 13.52 | 0.033200167 |
|  | BS00070791_51 | 7B | 314.71 | 0.00063 | 13.23 | 0.037758423 |
|  | Excalibur_c99967_87 | 3B | 256.15 | 0.0006483 | 13.17 | 0.039586513 |
|  | IAAV5776 | 1B | 506.14 | 0.0008645 | 12.53 | 0.041466668 |
|  | Ex_c13213_2992 | 2B | 371.86 | 0.0009798 | 12.25 | 0.046226388 |
| Grain Surface Area | D_contig57523_172 | 2D | 183.76 | 2.19E-05 | 23.19 | 0.012265047 |
|  | TA001185-0966 | 7D | 309.36 | 3.62E-05 | 22.00 | 0.012265047 |
|  | BobWhite_rep_c64004_321 | 2B | 461.38 | 0.0001551 | 18.65 | 0.024165848 |
|  | BS00035732_51 | 7D | 317.99 | 0.0001595 | 18.58 | 0.024165848 |
|  | D_GDS7LZN01CWBG5_74 | 7D | 317.99 | 0.0001595 | 18.58 | 0.031201339 |
|  | BobWhite_c9249_564 | 3A | 135.85 | 0.0002096 | 17.97 | 0.031635146 |
|  | Tdurum_contig58293_437 | 5B | 461.54 | 0.000214 | 17.92 | 0.031635146 |
|  | Ku_c47803_245 | 7D | 329.7 | 0.0002289 | 17.77 | 0.032073906 |
|  | BS00023035_51 | 4B | 238.43 | 0.0002575 | 17.51 | 0.044958956 |
|  | Kukri_c27958_334 | 6A | 153.27 | 0.0002685 | 17.42 | 0.044958956 |
|  | Kukri_c7241_322 | 4B | 237.18 | 0.0004012 | 16.53 | 0.044958956 |
|  | Kukri_c31502_115 | 6A | 190.27 | 0.0004184 | 16.44 | 0.044958956 |
|  | Tdurum_contig28316_442 | 1B | 226.95 | 0.0004336 | 16.36 | 0.044958956 |
|  | BS00022610_51 | 7D | 332.18 | 0.0004432 | 16.31 | 0.044958956 |
|  | Tdurum_contig48366_1324 | 4B | 237.18 | 0.0004914 | 16.08 | 0.044958956 |
|  | BobWhite_c10832_972 | 6B | 388.21 | 0.0006175 | 15.59 | 0.044958956 |
|  | Excalibur_c33002_123 | 7D | 375.36 | 0.0006185 | 15.58 | 0.044958956 |
|  | D_contig66051_204 | 1D | 131.49 | 0.0006637 | 15.43 | 0.044958956 |
|  | D_GBF1XID02IP0NJ_181 | 7D | 211.9 | 0.0007192 | 15.26 | 0.044958956 |
|  | IACX419 | 6B | 237.57 | 0.0009256 | 14.71 | 0.044958956 |
|  | Tdurum_contig45927_968 | 5A | 626.85 | 0.0009385 | 14.68 | 0.044958956 |
|  | Tdurum_contig64286_268 | 2A | 517.73 | 0.0009445 | 14.67 | 0.047288448 |
| Thousand grain weight | Tdurum_contig58293_437 | 5B | 461.54 | 3.03E-05 | 20.23 | 9.06E-05 |
|  | Tdurum_contig97656_120 | 3B | 136.78 | 3.55E-05 | 19.85 | 0.000109563 |
|  | Excalibur_c30178_439 | 5A | 98.8 | 0.0001457 | 16.51 | 0.000109563 |
|  | Tdurum_contig98477_350 | 5B | 461.54 | 0.0002606 | 15.17 | 0.000109563 |
|  | BobWhite_c5430_380 | 3B | 352.25 | 0.0002606 | 15.17 | 0.000109563 |
|  | Ku_c48337_2091 | 7D | 437.58 | 0.0002606 | 15.17 | 0.000117929 |
|  | RAC875_c8878_232 | 1B | 228.43 | 0.0002606 | 15.17 | 0.000137515 |
|  | wsnp_Ra_rep_c109853_92677055 | 2B | 351.23 | 0.0002606 | 15.17 | 0.000143082 |
|  | Excalibur_c37468_736 | 5A | 77.97 | 0.0003065 | 14.79 | 0.000143082 |
|  | BS00023142_51 | 1B | 227.17 | 0.0003106 | 14.76 | 0.00015267 |
|  | wsnp_Ex_rep_c104125_88923836 | 3A | 551.54 | 0.0003213 | 14.69 | 0.000155231 |
|  | Excalibur_c33040_447 | 2A | 463.74 | 0.0003344 | 14.60 | 0.000155231 |
|  | Kukri_c22602_1076 | 4A | 603.44 | 0.0003809 | 14.30 | 0.000193052 |
|  | Kukri_rep_c69810_502 | 1B | 226.95 | 0.0003809 | 14.30 | 0.00021067 |
|  | Tdurum_contig25853_348 | 2B | 351.23 | 0.0003809 | 14.30 | 0.000259117 |
|  | BobWhite_rep_c49533_93 | 1B | 549.23 | 0.0003809 | 14.30 | 0.000305673 |
|  | Ku_c1255_1107 | 3A | 433.76 | 0.0003809 | 14.30 | 0.000336191 |
|  | RAC875_c33470_345 | 1A | 108.79 | 0.0003809 | 14.30 | 0.000336191 |
|  | Tdurum_contig70846_226 | 1B | 220.23 | 0.0003809 | 14.30 | 0.000481906 |
|  | wsnp_Ex_rep_c67840_66538714 | 2B | 341.73 | 0.0003809 | 14.30 | 0.000577379 |
|  | Kukri_c7658_229 | 3D | 416.72 | 0.0004991 | 13.69 | 0.000645147 |
|  | Excalibur_c94962_57 | 3A | 551.54 | 0.0005457 | 13.49 | 0.000895949 |
|  | Tdurum_contig7529_227 | 2B | 339.63 | 0.0006839 | 12.99 | 0.000988729 |
|  | Kukri_rep_c111174_132 | 1B | 546.25 | 0.0007897 | 12.67 | 0.001061661 |
|  | IACX6214 | 3B | 134.89 | 0.0008978 | 12.38 | 0.001069176 |
|  | BS00023035_51 | 4B | 238.43 | 0.0009088 | 12.36 | 0.001069176 |
|  | RAC875_rep_c91630_132 | 5B | 401.49 | 0.0009257 | 12.32 | 0.001069176 |
|  | Ku_c9596_1649 | 3B | 113.62 | 0.0009706 | 12.21 | 0.001069176 |
| Grain yield per plant | RAC875_s119811_122 | 4A | 597.71 | 1.91E-05 | 21.36 | 0.017510726 |
|  | Tdurum_contig30286_549 | 6A | 143.43 | 3.42E-05 | 19.94 | 0.020703921 |
|  | BS00009674_51 | 4A | 597.71 | 3.99E-05 | 19.57 | 0.022241228 |
|  | Kukri_c1911_222 | 4A | 603.44 | 6.11E-05 | 18.55 | 0.023033053 |
|  | Ku_c2972_1754 | 3D | 11.67 | 0.0001702 | 16.15 | 0.023033053 |
|  | Excalibur_c14212_372 | 2B | 431.19 | 0.0001859 | 15.94 | 0.023120104 |
|  | IAAV1943 | 4A | 564.64 | 0.0001885 | 15.91 | 0.023154522 |
|  | Kukri_rep_c108973_667 | 5D | 523.54 | 0.0001897 | 15.89 | 0.027515389 |
|  | Tdurum_contig97656_120 | 3B | 136.78 | 0.0002087 | 15.67 | 0.029747157 |
|  | Excalibur_c7051_1115 | 2B | 444.88 | 0.0003821 | 14.29 | 0.03206866 |
|  | Ra_c31292_886 | 7D | 379.09 | 0.0004165 | 14.10 | 0.033200167 |
|  | RAC875_c53438_272 | 7B | 231.37 | 0.0004165 | 14.10 | 0.033200167 |
|  | Excalibur_c41557_147 | 3A | 284.63 | 0.0004466 | 13.94 | 0.037758423 |
|  | RAC875_c31606_234 | 2B | 429.47 | 0.0004681 | 13.84 | 0.039586513 |
|  | GENE-1657_48 | 3A | 284.63 | 0.0005593 | 13.44 | 0.041466668 |
|  | Tdurum_contig28085_87 | 7B | 236.99 | 0.000626 | 13.18 | 0.046226388 |
|  | wsnp_Ra_c6374_11143280 | 5B | 443.02 | 0.0006777 | 13.01 | 0.046390497 |
|  | RAC875_c97462_415 | 2B | 431.19 | 0.0008052 | 12.63 | 0.047673229 |
|  | RAC875_c50023_123 | 4A | 191.56 | 0.0008945 | 12.39 | 0.047673229 |
|  | Tdurum_contig49532_53 | 2B | 429.47 | 0.0009154 | 12.34 | 0.047673229 |

**Table S4: GWAS under Heat stressed Conditions**

| Trait | SNP | Chromosome | Position | P.value | R% | FDR_Adjusted_P-values |
| --- | --- | --- | --- | --- | --- | --- |
| Grain length | Excalibur_c22896_149 | 4A | 475.83 | 3.96E-06 | 25.36 | 0.003196545 |
|  | RAC875_c7734_411 | 4A | 497.16 | 9.25E-06 | 23.22 | 0.003342721 |
|  | RFL_Contig4686_700 | 4A | 475.83 | 1.18E-05 | 22.62 | 0.005028639 |
|  | Ex_c10574_1027 | 2D | 275.13 | 2.95E-05 | 20.37 | 0.005621909 |
|  | GENE-3548_384 | 2D | 193.66 | 4.73E-05 | 19.23 | 0.006874582 |
|  | wsnp_BG313770B_Ta_1_3 | 4A | 497.16 | 6.47E-05 | 18.48 | 0.008620596 |
|  | GENE-4708_214 | 7D | 150.76 | 0.000118 | 17.07 | 0.010760628 |
|  | BS00093856_51 | 3D | 72.19 | 0.000121 | 17.01 | 0.013310231 |
|  | Excalibur_c19666_203 | 4A | 475.83 | 0.000129 | 16.87 | 0.015245581 |
|  | RFL_Contig471_835 | 7D | 309.36 | 0.000131 | 16.82 | 0.016717607 |
|  | GENE-4937_537 | 2D | 298.38 | 0.000149 | 16.53 | 0.017510726 |
|  | Excalibur_c53131_187 | 3A | 276.05 | 0.000295 | 14.96 | 0.020703921 |
|  | Kukri_c17754_1179 | 4A | 475.83 | 0.000317 | 14.79 | 0.022241228 |
|  | Tdurum_contig5094_505 | 2A | 372.66 | 0.000324 | 14.74 | 0.023033053 |
|  | Tdurum_contig29515_467 | 4B | 163.94 | 0.000752 | 12.85 | 0.023033053 |
|  | Tdurum_contig12838_304 | 4A | 475.42 | 0.000823 | 12.65 | 0.023120104 |
|  | wsnp_BE444846B_Ta_2_2 | 1B | 208.49 | 0.000867 | 12.54 | 0.023154522 |
|  | Kukri_c11154_1723 | 5B | 126.02 | 0.000952 | 12.33 | 0.027515389 |
| Grain Width | BS00022027_51 | 1D | 108.87 | 1.21E-05 | 22.48 | 0.001069176 |
|  | Ra_c11738_1041 | 5A | 77.77 | 0.000184 | 15.97 | 0.001069176 |
|  | BobWhite_rep_c65565_359 | 1D | 108.87 | 0.000196 | 15.82 | 0.001069176 |
|  | Tdurum_contig29645_706 | 4B | 195.17 | 0.000204 | 15.73 | 0.001069176 |
|  | Excalibur_c35903_348 | 7B | 213.28 | 0.000327 | 14.65 | 0.001069176 |
|  | Kukri_rep_c109150_333 | 4B | 173.63 | 0.000343 | 14.54 | 0.001069176 |
|  | wsnp_CAP8_c1530_885118 | 6B | 218.86 | 0.000521 | 13.59 | 0.001069176 |
|  | Tdurum_contig5611_1064 | 3A | 280.36 | 0.000543 | 13.50 | 0.001136333 |
|  | Tdurum_contig6153_192 | 4B | 173.63 | 0.000565 | 13.41 | 0.001136333 |
|  | Kukri_c20197_1608 | 7B | 213.28 | 0.000612 | 13.24 | 0.001136333 |
|  | D_contig36392_363 | 3D | 454.76 | 0.000629 | 13.17 | 0.00220335 |
|  | Tdurum_contig54776_1489 | 4A | 641.89 | 0.00074 | 12.81 | 0.002953831 |
|  | Excalibur_c54967_930 | 7B | 213.28 | 0.000775 | 12.71 | 0.003196545 |
|  | CAP7_rep_c9997_155 | 1D | 108.87 | 0.000863 | 12.47 | 0.003342721 |
|  | Tdurum_contig59755_643 | 7B | 427.7 | 0.000871 | 12.45 | 0.005028639 |
|  | Excalibur_rep_c103202_402 | 4B | 243.79 | 0.000989 | 12.17 | 0.005621909 |
| Grain Diameter | Tdurum_contig14863_885 | 5A | 67.48 | 3.88E-06 | 29.88 | 0.029747157 |
|  | BobWhite_c836_422 | 5D | 162.12 | 6.36E-06 | 28.68 | 0.03206866 |
|  | BobWhite_rep_c61300_88 | 7B | 206.22 | 1.04E-05 | 27.51 | 0.033200167 |
|  | Excalibur_c20796_395 | 7A | 372.34 | 3.11E-05 | 24.94 | 0.033200167 |
|  | Kukri_c5166_994 | 3B | 229.68 | 3.88E-05 | 24.43 | 0.037758423 |
|  | Kukri_rep_c87210_361 | 5A | 77.82 | 5.17E-05 | 23.77 | 0.039586513 |
|  | Tdurum_contig1015_131 | 3D | 292.51 | 6.23E-05 | 23.35 | 0.041466668 |
|  | Tdurum_contig46313_394 | 4B | 211.4 | 6.68E-05 | 23.19 | 0.046226388 |
|  | Ex_c8261_2309 | 3D | 416.72 | 9.83E-05 | 22.32 | 0.046390497 |
|  | Excalibur_c1895_826 | 4A | 430.71 | 0.000234 | 20.39 | 0.037758423 |
|  | BS00010868_51 | 1B | 35.47 | 0.000258 | 20.18 | 0.039586513 |
|  | Kukri_c58004_937 | 3B | 45.85 | 0.000269 | 20.08 | 0.003196545 |
|  | Tdurum_contig15645_1255 | 7D | 298.3 | 0.000279 | 20.01 | 0.003342721 |
|  | RAC875_c64610_285 | 7D | 358.28 | 0.000521 | 18.66 | 0.005028639 |
|  | BS00010616_51 | 7B | 186.24 | 0.000596 | 18.37 | 0.005621909 |
|  | Excalibur_c27873_266 | 1D | 268.67 | 0.000787 | 17.78 | 0.006874582 |
|  | wsnp_Ex_c24135_33382700 | 2B | 368.81 | 0.000811 | 17.72 | 0.008620596 |
|  | Kukri_c38985_537 | 2B | 64.65 | 0.000881 | 17.54 | 0.010760628 |
|  | Kukri_c99538_516 | 7B | 223.87 | 0.000897 | 17.50 | 0.013310231 |
|  | Kukri_c23474_718 | 3D | 283.69 | 0.000901 | 17.49 | 0.015245581 |
| Grain Roundness | D_GB5Y7FA02JIMB5_49 | 7D | 290.6 | 1.02E-07 | 35.01 | 0.00197205 |
|  | BS00093856_51 | 3D | 72.19 | 4.25E-07 | 31.12 | 0.005464054 |
|  | RAC875_rep_c106337_414 | 5B | 223.52 | 9.99E-07 | 28.86 | 0.008509816 |
|  | GENE-4937_537 | 2D | 298.38 | 1.10E-06 | 28.59 | 0.008509816 |
|  | Tdurum_contig86243_288 | 2A | 479.11 | 4.37E-06 | 25.04 | 0.018711612 |
|  | Ex_c56664_126 | 7A | 635.34 | 1.73E-05 | 21.61 | 0.015245581 |
|  | wsnp_Ra_rep_c74879_72651462 | 4B | 279.05 | 2.27E-05 | 20.94 | 0.016717607 |
|  | D_contig01033_757 | 4A | 176.48 | 3.35E-05 | 19.99 | 0.017510726 |
|  | D_contig19866_324 | 7D | 211.9 | 3.83E-05 | 19.67 | 0.020703921 |
|  | D_F1BEJMU01A6MWB_163 | 3B | 551.3 | 0.000146 | 16.50 | 0.022241228 |
|  | Ex_c10574_1027 | 2D | 275.13 | 0.000178 | 16.04 | 0.023033053 |
|  | RAC875_c47741_227 | 1B | 311.35 | 0.000328 | 14.64 | 0.023033053 |
|  | Kukri_c48_1058 | 3D | 286.14 | 0.000377 | 14.32 | 0.023120104 |
|  | RAC875_rep_c89232_448 | 5D | 311.73 | 0.00041 | 14.13 | 0.023154522 |
|  | Tdurum_contig49608_1323 | 4B | 45.2 | 0.00041 | 14.13 | 0.027515389 |
|  | wsnp_Ku_c8927_15048149 | 2A | 388.71 | 0.000469 | 13.83 | 0.029747157 |
|  | D_contig24848_84 | 6D | 295.74 | 0.000639 | 13.14 | 0.03206866 |
|  | Excalibur_c5346_2716 | 7D | 390.46 | 0.000673 | 13.02 | 0.033200167 |
|  | Excalibur_c31131_64 | 7B | 213.28 | 0.000701 | 12.93 | 0.033200167 |
|  | CAP11_c2329_154 | 4A | 597.71 | 0.000807 | 12.62 | 0.037758423 |
|  | tplb0040n03_560 | 4A | 185.89 | 0.000818 | 12.59 | 0.039586513 |
|  | BS00070050_51 | 2B | 104.46 | 0.000819 | 12.59 | 0.033200167 |
|  | Tdurum_contig30877_235 | 1B | 304.37 | 0.000934 | 12.30 | 0.033200167 |
|  | Excalibur_c17557_72 | 2B | 346.46 | 0.000979 | 12.19 | 0.037758423 |
|  | Ex_c10068_1509 | 2A | 479.11 | 0.000986 | 12.18 | 0.039586513 |
| Grain Circumferences | D_contig57523_172 | 2D | 183.76 | 4.61E-07 | 31.12 | 0.000162707 |
|  | Tdurum_contig28055_176 | 7B | 32.21 | 5.58E-06 | 24.65 | 0.000208403 |
|  | RFL_Contig801_2124 | 7B | 32.21 | 9.84E-06 | 23.23 | 0.000208403 |
|  | Tdurum_contig25857_274 | 7B | 32.21 | 9.84E-06 | 23.23 | 0.000208403 |
|  | Kukri_c27958_334 | 6A | 153.27 | 1.01E-05 | 23.18 | 0.000208403 |
|  | Excalibur_rep_c109282_92 | 2B | 311.02 | 5.13E-05 | 19.22 | 0.000208403 |
|  | RAC875_c5103_2161 | 5D | 161.88 | 5.23E-05 | 19.17 | 0.000208403 |
|  | Excalibur_c25991_184 | 7B | 169.41 | 5.57E-05 | 19.02 | 0.000208403 |
|  | RAC875_c37592_426 | 7A | 103.7 | 9.48E-05 | 17.76 | 0.000208403 |
|  | D_contig22507_191 | 6D | 191.27 | 0.000159 | 16.56 | 0.000208403 |
|  | D_contig29746_525 | 7D | 436.21 | 0.000159 | 16.56 | 0.000208403 |
|  | D_GCE8AKX01CTXDI_46 | 6D | 191.27 | 0.000159 | 16.56 | 0.000208403 |
|  | Ex_c6013_558 | 5D | 185.6 | 0.000159 | 16.56 | 0.000208403 |
|  | Ex_c7795_2122 | 2B | 450.63 | 0.000159 | 16.56 | 0.000208403 |
|  | Excalibur_c16743_171 | 2B | 311.02 | 0.000159 | 16.56 | 0.000208403 |
|  | Excalibur_c22503_590 | 3D | 432.46 | 0.000159 | 16.56 | 0.000208403 |
|  | RAC875_rep_c77067_347 | 3A | 67.13 | 0.000942 | 12.55 | 0.000208403 |
|  | RAC875_rep_c96433_140 | 5B | 585.84 | 0.000942 | 12.55 | 0.000208403 |
|  | Tdurum_contig27634_93 | 7B | 166.48 | 0.000964 | 12.50 | 0.000208403 |
|  | Excalibur_c63243_316 | 1A | 216.46 | 0.000972 | 12.48 | 0.000208403 |
|  | Excalibur_c7713_272 | 6B | 312.5 | 0.000978 | 12.46 | 0.003633157 |
| Grain Surface Area | Excalibur_rep_c69263_462 | 4B | 182.55 | 6.09E-06 | 34.88 | 0.000481906 |
|  | RAC875_c28057_144 | 7B | 61.42 | 7.48E-06 | 34.44 | 0.000577379 |
|  | BS00072264_51 | 5A | 124.08 | 1.43E-05 | 33.05 | 0.000645147 |
|  | Excalibur_c3423_994 | 7B | 77 | 1.43E-05 | 33.05 | 0.000895949 |
|  | Ku_c10135_987 | 5A | 78.15 | 1.43E-05 | 33.05 | 0.000988729 |
|  | wsnp_Ra_c3270_6136601 | 1A | 256 | 0.000205 | 27.59 | 0.001061661 |
|  | BS00103846_51 | 7A | 410.68 | 0.000208 | 27.56 | 0.001069176 |
|  | RAC875_c5893_368 | 6A | 338.95 | 0.000235 | 27.32 | 0.001069176 |
|  | D_GDEEGVY01B1RNW_44 | 5B | 312.66 | 0.00026 | 27.13 | 0.001069176 |
|  | IACX5793 | 1A | 256 | 0.000291 | 26.90 | 0.001069176 |
|  | BS00108780_51 | 7A | 372.34 | 0.000304 | 26.82 | 0.001069176 |
|  | Tdurum_contig45661_684 | 2A | 465.15 | 0.000315 | 26.75 | 0.001069176 |
|  | wsnp_Ex_c16018_24438963 | 5A | 709.71 | 0.000389 | 26.34 | 0.001069176 |
|  | BS00011224_51 | 4A | 222.92 | 0.000675 | 25.27 | 0.001069176 |
|  | Tdurum_contig12084_1122 | 7B | 195.74 | 0.000675 | 25.27 | 0.001069176 |
|  | IAAV4838 | 7A | 477.62 | 0.000682 | 25.25 | 0.001069176 |
|  | Excalibur_c88370_144 | 7D | 50.6 | 0.000703 | 25.20 | 0.001069176 |
|  | D_contig36054_1409 | 1A | 66.54 | 0.000713 | 25.17 | 0.001136333 |
|  | wsnp_Ex_c27898_37058842 | 7A | 482.42 | 0.000735 | 25.11 | 0.001136333 |
|  | GENE-4403_405 | 7B | 244.31 | 0.000765 | 25.03 | 0.001136333 |
|  | Tdurum_contig93508_295 | 2A | 466.18 | 0.000778 | 25.00 | 0.00220335 |
|  | BS00100120_51 | 1A | 260.38 | 0.000845 | 24.85 | 0.002953831 |
| Thousand Grain Weight | Tdurum_contig44851_927 | 1B | 513.23 | 3.83E-07 | 29.88 | 0.003196545 |
|  | Tdurum_contig10105_484 | 3A | 271.98 | 6.18E-07 | 28.70 | 0.003342721 |
|  | RAC875_c53438_272 | 7B | 231.37 | 2.10E-06 | 25.75 | 0.005028639 |
|  | Ra_c31292_886 | 7D | 379.09 | 2.10E-06 | 25.75 | 0.005621909 |
|  | Tdurum_contig97206_869 | 4A | 138.18 | 4.16E-06 | 24.14 | 0.006874582 |
|  | RAC875_c61512_173 | 1B | 304.37 | 5.48E-06 | 23.50 | 0.008620596 |
|  | Excalibur_c31060_904 | 4A | 191.56 | 1.06E-05 | 21.97 | 0.010760628 |
|  | Tdurum_contig81288_341 | 7A | 132.86 | 2.73E-05 | 19.83 | 0.013310231 |
|  | RAC875_c28390_99 | 6A | 115.71 | 6.22E-05 | 18.01 | 0.015245581 |
|  | RFL_Contig2531_2094 | 4A | 564.64 | 6.31E-05 | 17.98 | 0.016717607 |
|  | Tdurum_contig10300_423 | 4B | 183.6 | 7.76E-05 | 17.52 | 0.017510726 |
|  | Excalibur_c12423_656 | 5D | 264.89 | 9.72E-05 | 17.04 | 0.020703921 |
|  | IAAV3545 | 4A | 564.64 | 0.000173 | 15.79 | 0.022241228 |
|  | Tdurum_contig11004_400 | 1A | 173.23 | 0.000187 | 15.63 | 0.023033053 |
|  | BobWhite_c18861_438 | 1A | 129.97 | 0.000208 | 15.40 | 0.023033053 |
|  | BobWhite_c28009_397 | 7D | 120.98 | 0.000219 | 15.29 | 0.023120104 |
|  | RAC875_c29060_307 | 4B | 182.55 | 0.000222 | 15.27 | 0.023154522 |
|  | Tdurum_contig9162_259 | 1B | 484.13 | 0.000301 | 14.62 | 0.027515389 |
|  | RFL_Contig3509_229 | 2A | 430.56 | 0.000319 | 14.50 | 0.029747157 |
|  | Ex_c27608_466 | 6D | 180.23 | 0.000513 | 13.50 | 0.03206866 |
|  | Excalibur_c9183_1397 | 7A | 123.58 | 0.000581 | 13.24 | 0.033200167 |
|  | BS00065808_51 | 2A | 447.29 | 0.000834 | 12.50 | 0.033200167 |
|  | Kukri_rep_c111174_132 | 1B | 546.25 | 0.000915 | 12.31 | 0.037758423 |
|  | Excalibur_c19552_319 | 3B | 278.84 | 0.000919 | 12.30 | 0.039586513 |
| Grain yield per Plant | D_contig24171_152 | 6D | 191.27 | 4.71E-06 | 28.74 | 0.024165848 |
|  | BS00063821_51 | 7B | 535.94 | 8.16E-06 | 27.55 | 0.024165848 |
|  | RFL_Contig3005_1138 | 7B | 535.94 | 8.16E-06 | 27.55 | 0.031201339 |
|  | Kukri_rep_c103009_826 | 6B | 80.92 | 8.94E-06 | 27.36 | 0.031635146 |
|  | Tdurum_contig85180_99 | 1B | 303.91 | 0.000114 | 22.05 | 0.031635146 |
|  | RAC875_c42365_61 | 1A | 216.46 | 0.000128 | 21.81 | 0.032073906 |
|  | Ku_c6546_718 | 2B | 312.78 | 0.000131 | 21.75 | 0.044958956 |
|  | Kukri_c20060_350 | 6D | 249.83 | 0.000137 | 21.67 | 0.044958956 |
|  | RAC875_c46661_184 | 2B | 312.78 | 0.000149 | 21.50 | 0.044958956 |
|  | RAC875_c26328_75 | 7B | 169.41 | 0.000159 | 21.37 | 0.044958956 |
|  | wsnp_Ex_rep_c66524_64798744 | 2B | 319.82 | 0.000163 | 21.33 | 0.044958956 |
|  | Excalibur_c16743_171 | 2B | 311.02 | 0.000163 | 21.33 | 0.044958956 |
|  | Ku_c181_790 | 3A | 218.69 | 0.000163 | 21.33 | 0.044958956 |
|  | Excalibur_c57840_227 | 6B | 341.97 | 0.00018 | 21.13 | 0.044958956 |
|  | Tdurum_contig38998_55 | 4A | 602.49 | 0.000192 | 20.99 | 0.044958956 |
|  | Excalibur_c25991_184 | 7B | 169.41 | 0.000195 | 20.96 | 0.044958956 |
|  | wsnp_Ex_c51352_55323092 | 2B | 312.26 | 0.000206 | 20.85 | 0.044958956 |
|  | BS00076402_51 | 7B | 32.21 | 0.000212 | 20.80 | 0.044958956 |
|  | D_contig33267_423 | 6B | 218.23 | 0.000215 | 20.77 | 0.044958956 |
|  | RAC875_c45591_79 | 7B | 246.47 | 0.000218 | 20.74 | 0.047288448 |
|  | Excalibur_c58468_162 | 7B | 418.37 | 0.000243 | 20.53 | 0.047288448 |
